# A Scoping Review of IPV Prevention Curricula and Their Implementation in Low- and Middle-Income Countries: Implications for the Next Generation of Programs

**DOI:** 10.3390/ijerph23080989

**Published:** 2026-07-29

**Authors:** Kate Doyle, Ruti G. Levtov, Lori Rolleri, Erin Stern, Lori Heise

**Affiliations:** 1 Prevention Collaborative, Ottawa, ON K2A 1C2, Canada; r.levtov@prevention-collaborative.org (R.G.L.); e.stern@prevention-collaborative.org (E.S.); l.heise@prevention-collaborative.org (L.H.); 2 Lori Rolleri Consulting, Durham, NC 27707, USA; lorirolleri@gmail.com

**Keywords:** intimate partner violence, violence against women, gender transformative, curriculum, violence prevention, program implementation, program adaptation, participatory learning, alcohol, mental health

## Abstract

**Highlights:**

**Public health relevance—How does this work relate to a public health issue?**

- Intimate partner violence (IPV) is a preventable global public health crisis: at least one in three women will experience physical or sexual violence from a male partner in their lifetime, with higher past-year prevalence in low- and middle-income countries (LMICs).
- Curriculum-based programs are among the most widely used strategies for preventing IPV in LMICs, yet very little is systematically known about how these programs compare in design, content, and implementation, limiting the field’s capacity to learn and improve.

**Public health significance—Why is this work of significance to public health?**

- This is the most comprehensive review of IPV prevention curricula in LMICs to date, examining 55 curricula from 42 programs across 23 countries. Uniquely, it focuses on program design, content, and implementation features rather than outcomes.
- The review identifies considerable variation in program design and implementation but similarity in core content, including widespread adaptation of existing programs and activities, revealing areas to strengthen and innovate in future programs.

**Public health implications—What are the key implications or messages for practitioners, policy makers and/or researchers in public health?**

- The field should prioritize continued innovation in new program models and content, while strengthening relationship skills-building, including communication and emotional regulation, and more intentionally addressing key risk factors like alcohol and mental health.
- Practitioners, program designers, and funders can support better design, planning, and implementation of programs grounded in evidence and good practice, and improve program documentation to support learning, intentional adaptation, and replication.

**Abstract:**

Curriculum-based programs are one of the most common and effective strategies for preventing intimate partner violence (IPV) in low- and middle-income countries (LMICs). Yet relatively little is known about how curricula and their implementation compare across programs, limiting learning and innovation. This scoping review examined the design, content, and implementation of 55 curricula from 42 programs implemented in 23 LMICs between 2001 and 2024. Curricula were identified through screening systematic reviews, grey literature, donor reports, and professional contacts. Data on program characteristics, subject matter, methodologies, and implementation were extracted, synthesized, and analyzed. We found curricula employed similar participatory methodologies and content, and violence, gender, relationships, power, and communication emerged as core topics. Adaptation of earlier programming was widespread: only five curricula did not cite content adapted from an earlier program. Key gaps included the relatively limited use of evidence-based strategies to build relationship skills and content addressing alcohol use and poor mental health, important IPV risk factors. Implementation varied considerably in terms of intensity, frequency, duration, group size, facilitators and their training, and information on some features was sparse. The findings highlight several opportunities for strengthening and innovation, for which we present six recommendations to guide future IPV prevention programming.

## 1. Introduction

Intimate partner violence (IPV), the most common form of violence women experience, is preventable [1,2]. Globally, at least one in three women will experience physical or sexual violence from a male partner in their lifetime [1]. Risk factors for IPV include poor mental health, harmful alcohol consumption, conflict, poverty, norms that promote male privilege and power, and the acceptance of violence [3]. Women and girls who experience IPV face numerous social, economic, and health consequences, including to their sexual and reproductive health and mental health [4]. Women and girls in certain contexts and among specific populations are more likely to experience IPV, and the past-year prevalence of IPV is greater in low- and middle-income countries (LMICs) than in high-income ones [1]. However, the last two decades have seen a significant growth in IPV prevention programs and evidence in LMICs [2].

Facilitated learning and reflection groups are one of the most common IPV prevention strategies [2,4]. These programs, sometimes referred to as group education or discussion groups, usually take a gender transformative approach, meaning they seek to actively challenge the underlying power structures and rigid gender norms that drive violence [5]. They use participatory and interactive learning methods to challenge gender-stereotyped ideas about men’s and women’s roles in society, engage women and men in critical reflection about gender and power, and build healthy relationship skills such as communication [4,6]. Such programs are usually guided by a curriculum designed to support consistent implementation [4].

There is robust evidence that gender transformative curriculum-based programs can prevent or reduce IPV across a range of contexts and populations [7]. Research suggests programs are most effective when they work intensively with men and women to enable in-depth reflection, learning and skills-building, are delivered by well-trained and supported personnel, and are guided by clear curricula that reflect a well-defined theory of change which addresses multiple risk factors for IPV [7,8]. A theory of change can be considered “a map for intervention design and evaluation impact, setting out how an intervention should bring about change.” [4] (p. 4). Despite greater evidence of their effectiveness, we know relatively little about how curriculum-based programs compare across countries, contexts, and populations. Published information on program design, curricula content, and implementation is often ‘thin’ or missing [4,9]. A better understanding of these features could support more effective programming and adaptation [7,10].

**Aim of this study**

This study aimed to improve our understanding of curriculum-based IPV prevention programs and their implementation in LMICs. The review sought to answer three broad research questions:

(1) what design features characterize IPV prevention curricula;
(2) what are their core content and learning methodologies; and
(3) how are curricula implemented and facilitated.

By answering these questions, the review aimed to identify opportunities to innovate and strengthen future curriculum-based IPV prevention programs.

## 2. Materials and Methods

We reviewed a wide range of IPV prevention curricula that guide facilitated learning and reflection groups across different countries and diverse populations. We followed scoping review guidelines and the PRISMA-ScR extension but modified our search strategy to reflect our focus on curricula themselves, rather than published studies about their effectiveness [11,12,13]. We designed a pragmatic search strategy, combining complementary approaches, to identify as many eligible programs as possible and reduce the likelihood of omitting relevant curricula. The study protocol was retrospectively registered on Open Science Framework (OSF) [14]. Supplementary File S1 includes more details on the search strategy and Supplementary File S2 includes the PRISMA-ScR checklist.

The study also draws on relevant data and insights from an earlier stage of the project, which sought to review IPV prevention curricula and link their characteristics and implementation to evaluated impact. The project found that implementation was inconsistently or sparsely reported across include curricula, limiting the ability to draw such connections. This gap shaped one of the objectives of the current review, to better understand implementation, and informed a revised search strategy.

### 2.1. Inclusion and Exclusion Criteria

Our search occurred in two stages. We first set out to identify programs that aimed to prevent IPV and included a facilitated group component and then sought to obtain their curricula. The inclusion criteria were:

(a) Program was designed with an explicit goal of preventing IPV, as opposed to other forms of violence against women and girls (e.g., non-partner sexual violence or sexual harassment);
(b) Implemented in a low- or middle-income country, as our organization focuses on supporting violence prevention practitioners in these countries;
(c) Engaged participants in facilitated group sessions (with three or more participants) guided by a structured curriculum;
(d) Implemented in 2000 or later given the growth in violence prevention programming and evidence since then; and
(e) Curriculum available for review in English, French, or Spanish.

Programs with additional goals, such as HIV prevention or economic empowerment, were included if IPV prevention was also an explicit aim. There were no age restrictions for participants. We defined a ‘curriculum’ as a document containing an instructional plan, content, and subject matter designed to guide the facilitation of group sessions, which is expected to be implemented with reasonable fidelity [4]. Curricula combining individual and group sessions were eligible.

We excluded manuals that were:

(a) not intended for full implementation with the same participants (e.g., a compendium of optional activities often used in community mobilization programs); or
(b) designed to guide the training of program facilitators.

### 2.2. Curricula Identification and Selection

**Figure 1:**
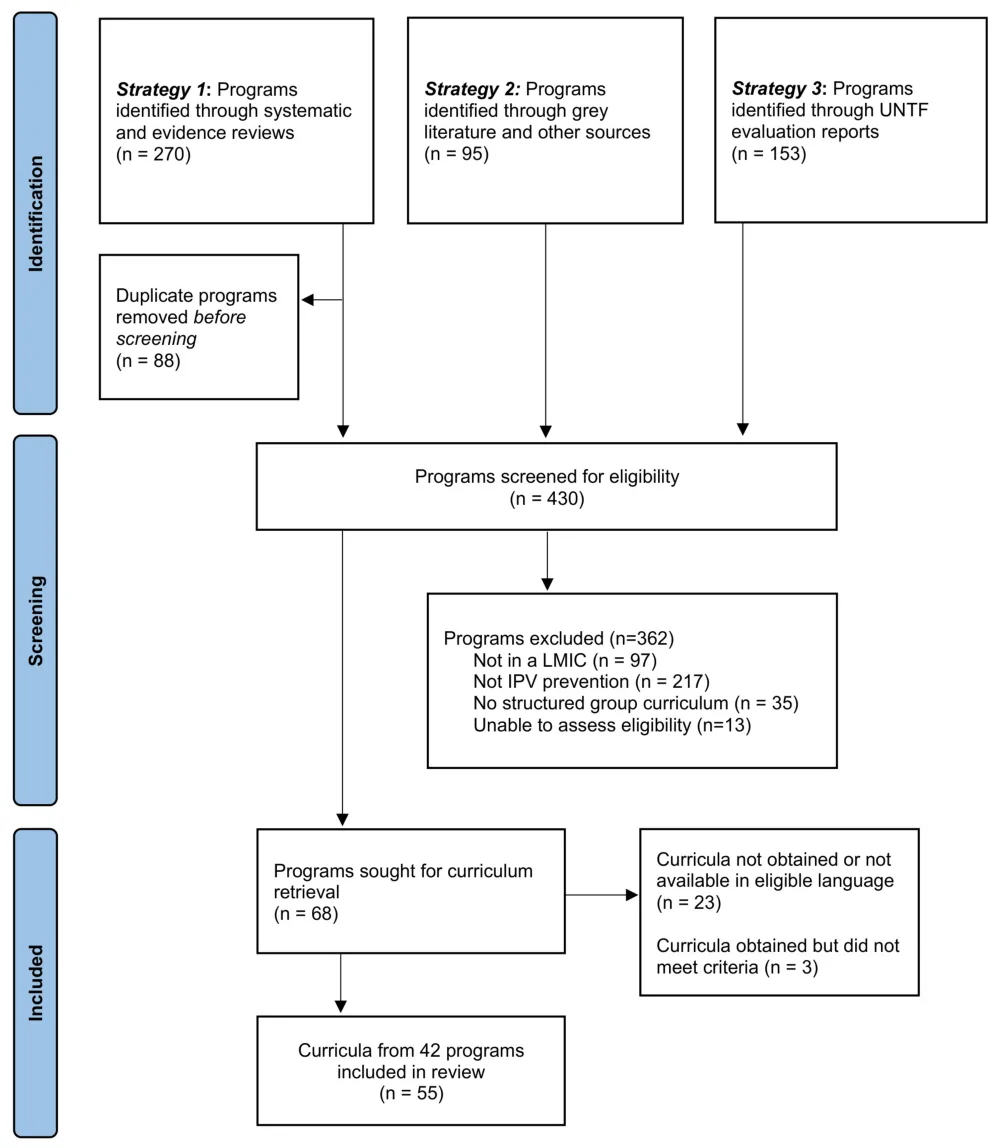
Curricula identification and selection process.

We used several strategies to identify eligible programs, as depicted in Figure 1, and conducted the search between January 2024 and April 2025. This allowed newer curricula to be included as they were made available. First, we consulted eight systematic or evidence reviews published since 2020 that included violence prevention programs in LMICs [7,9,10,15,16,17,18,19]. After removing duplicates, these reviews cited 182 unique programs, which we screened for eligibility using information in the reviews or original publications, yielding 35 eligible programs. Second, we assessed 95 additional programs identified through grey literature, professional contacts, or violence prevention funder and implementation websites, from which we identified 17 additional eligible programs.

Third, we consulted 153 publicly available evaluation reports for projects funded by the United Nations Trust Fund to End Violence against Women and Girls (UN Trust Fund), which grants to organizations preventing and responding to violence [20]. We did this to identify programs that might not be captured in the published literature, including those implemented by smaller non-governmental organizations. Many projects had multiple components, and it was not always possible to determine whether these included a curriculum-based component from the reports alone. Where feasible, UN Trust Fund staff connected us with program implementers to further assess eligibility. After screening, we identified 16 additional programs.

In total, we screened 430 programs and identified 68 meeting our criteria. Curricula were obtained from 45 programs through web searches or by contacting program developers or implementers. Some programs included multiple curricula designed for different populations. Three programs were later excluded because they did not meet our definition of a curriculum. Ultimately, the study includes 55 curricula from 42 programs. For each curriculum, we also collected any available documentation on implementation, such as program reports, evaluations, facilitator guidance, or training manuals.

All authors were involved in identifying eligible curricula. The first author led the screening of evidence reviews and UN Trust Fund reports, and the initial review of curricula, and consulted another author if eligibility was unclear.

### 2.3. Data Extraction

After downloading documents, we assigned curricula to four categories based on the main goals of the program in which they were implemented, as some programs sought to prevent IPV alongside other primary goals. Program categories included: (1) violence against women and girls; (2) sexual and reproductive health (SRH); (3) economic strengthening, which sought to advance household economic security or women’s economic empowerment; and (4) family strengthening, which sought to advance family or child wellbeing.

We categorized programs based on program implementers’ descriptions of each program’s goals and components. Some programs included multiple curricula, each designed for a different population and sometimes with differing aims; we categorized each program by its overall aim. Programs with no stated aim beyond violence prevention were categorized as violence against women and girls programs. Several programs spanned multiple categories; to aid in analysis of program differences, we limited each to a single category. Specifically, three programs had stated aims in both economic strengthening and SRH. We categorized these as economic strengthening programs because they were built around distinct components, such as microfinance, savings groups, or financial training. As a result, the design, content, and implementation of their curricula likely differed from other SRH programs.

Data on program design and implementation characteristics were extracted from curricula and their supporting documents and recorded systematically in Microsoft Excel for Mac, version 16.80 (Microsoft Corporation, Redmond, WA, USA). See Supplementary File S3 for a full list of documents. Extracted data included program characteristics, for example, country, partners, goals, and implementation details, such as participant characteristics, total number and frequency of sessions, hours of instruction, and facilitator characteristics. Subjective reviewer impressions on curriculum content and implementation (strengths, possible weaknesses, unique attributes) were also recorded.

Data were cross-checked across documents, and reports describing actual implementation were prioritized. Where feasible, we reached out to program implementers to clarify aspects of implementation or conducted web searches to obtain more information. Insights on the curricula teaching and learning methodologies captured during an earlier stage of the project, which included 17 of the 55 curricula included in the final review, were also verified and integrated where relevant.

Data on curricula content and learning methodologies were extracted directly from curricula. A document sometimes included distinct instruction plans and subject matter for facilitating different groups (e.g., men’s groups, women’s groups); these were reviewed as two separate curricula. Several programs included economic and social empowerment curricula developed in tandem (i.e., integrating gender or violence prevention content within the former); our review considered the two as a single curriculum.

The first and second authors reviewed curricula and recorded data on subject matter addressed in a second charting table. Topic areas were identified through an iterative process combining deductive and inductive approaches. A preliminary list of topics (codes) was developed in advance, based on existing knowledge of common curricula content, topics identified during an earlier stage of the project, and key risk and protective factors for IPV. Additional codes were added as new topics emerged. After an initial review by the first author, the second author reviewed a random sample of 20% of the curricula to assess consistency in how topics were coded. The charting process and codes were then discussed and refined by both authors. The charting captured whether a curriculum included a given topic area, which had to be a key focus or objective of the session and not just mentioned or implied within discussion questions, and the number of sessions addressing that topic. Multiple topics were possible per session.

Ultimately, each curriculum was reviewed at least three times to validate the coding. Optional content or activities, and background resources for facilitators were not coded. For the two programs that included individual and group sessions, we coded the content from both types of sessions. In a few instances, software was used to translate limited portions of curricula content that were not in English, French, or Spanish (e.g., text in visuals or case studies).

Throughout the data extraction and charting process, the authors held regular meetings to discuss progress, address questions or discrepancies, and refine the approach. When an assessment was unclear or there was disagreement between reviewers, consensus was reached through discussion.

### 2.4. Analysis

Numeric and qualitative data extracted from curricula and supporting documents were summarized and analyzed to answer our research questions. Descriptive statistics, such as frequencies and proportions, were used to identify and report patterns in program design, curricula content, and implementation characteristics, including by program type and population.

Following data extraction and analysis, we engaged in an ongoing process to synthesize and interpret the findings in relation to the broader evidence base [21]. This involved structured team discussions in which the extracted data were considered alongside the existing literature, including assessing the extent to which the reviewed programs reflected evidence-based practices. We also engaged in the consultation phase proposed by Arksey and O’Malley, holding multiple meetings with violence prevention practitioners at different stages of the review, to describe our methodology, share findings, and discuss their applicability to the field [11,21]. All authors discussed and validated the overarching findings and their practical implications for the field presented in this paper.

## 3. Results

We reviewed 55 curricula designed to guide facilitated learning and reflection groups from 42 programs, implemented in 23 countries between 2001 and 2024. The results section presents: the characteristics of reviewed curricula (Section 3.1); findings on curricula content and subject matter (Section 3.2); findings on curricula teaching approaches, learning methods, and adaptation (Section 3.3); and findings on implementation and facilitation (Section 3.4).

### 3.1. Characteristics of Reviewed Curricula

**Figure 2:**
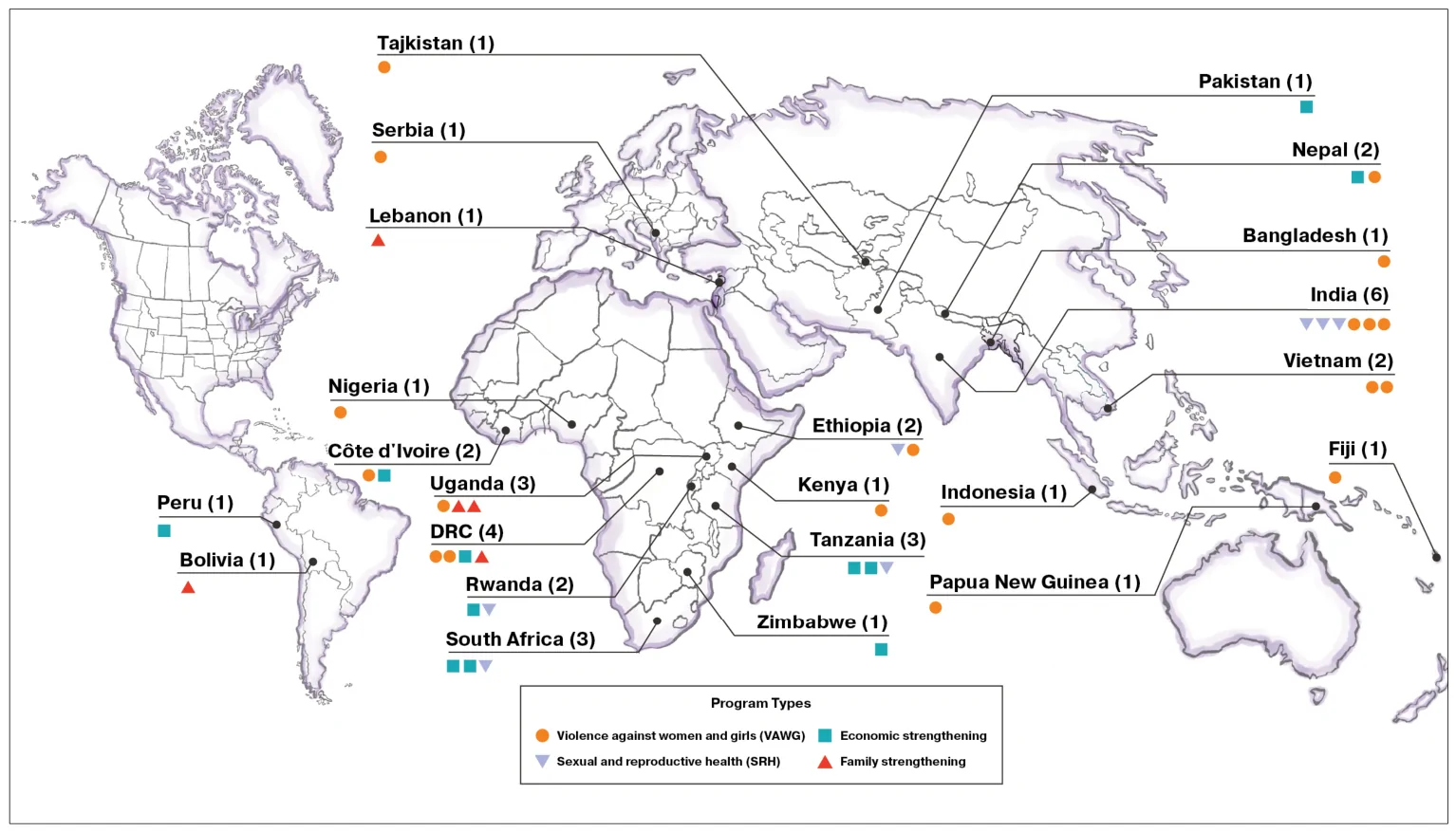
Geographic distribution of programs by country (number of programs in parenthesis). Image copyright: Prevention Collaborative, 2026. Used with permission.

Table 1 captures key characteristics of reviewed curricula, while Supplemental Table S1 provides details for each program. Half the curricula (51%) were implemented in Sub-Saharan Africa and slightly more than a quarter in South Asia. There was more limited representation from other regions (See Figure 2). Programs were implemented in a variety of contexts, primarily in rural (40%) or urban (38%) areas.

#### 3.1.1. Program Goals and Implementers

All the reviewed curricula were designed to prevent IPV, but slightly less than half came from programs whose sole purpose was to prevent violence against women and girls (VAWG). The remaining curricula (55%) were implemented in programs that sought to prevent IPV alongside other primary goals, including SRH (24%), economic strengthening (18%), or family strengthening programs (13%). Most curricula (80%) were implemented alongside other program components, such as village savings and loans associations (VSLAs), community mobilization activities, or social behavior change campaigns. Some, but not all, programs included facilitated learning and reflection groups designed for different populations.

Curricula were primarily developed and implemented by international non-governmental organizations (NGOs) (38%), by national or local NGOs or universities (26%), or through partnerships between national NGOs and international NGOs or universities (36%).

#### 3.1.2. Populations

Curricula were designed for implementation with a wide variety of populations, including boys, girls, men, and women, aged 11 to 80 years, often in lower-income communities. More than half the curricula (54.5%) were designed for men and women (or boys and girls), although some programs reached men and women separately, which we explore under implementation findings. The majority were delivered to adults aged 18 years or older (78%), followed by adolescents and adults (mostly young adults) (13%), or only adolescents (9%). Some curricula were designed to engage a range of community members (more common in VAWG programs), while others sought to reach specific sub-populations, such as savings group members or newly married couples.

Nearly half of the curricula (45.5%) were designed for married or partnered individuals, although both partners were not always engaged in the change process. Eight curricula (14%) were designed for parents of (mostly) young children, six of which had a particular focus on fathers. Several programs worked intergenerationally, engaging participants’ children, parents, or in-laws in specific sessions. A few programs were designed to reach groups at higher risk of violence and/or HIV, including two for refugees and displaced persons and one for female sex workers and their partners.

### 3.2. Findings on Curricula Content and Subject Matter

This section begins with findings on how curricula introduced learning goals to participants, followed by the topic areas covered, including common activities and their origins. Table 2 presents 18 topic areas, defined in Supplemental Table S2. For each topic, we assessed whether it was included in a curriculum, the number of sessions addressing it, and the proportion of total sessions this represented. This captures the breadth and focus of curricula content, but not the depth or quality with which topics were addressed. Where notable differences existed across programs or populations, these are described in the text, with additional details in Supplemental Tables S3 and S4.

#### 3.2.1. Curricula Often Used Aspirational Messaging to Highlight the Benefits of Healthier Relationships for Individuals, Families, and Communities

Curricula commonly employed aspirational messaging to convey that change is possible and to highlight the benefits of program participation. Programs described themselves as supporting participants through a journey or process of change, growth, empowerment, or transformation. For example, helping participants to reflect on their personal goals and look within themselves to consider what needs to change to achieve them. Participants were told that participation could bring benefits such as improved health, economic security, and stronger family or partner relationships. Relationships were described as becoming healthier, happier, stronger, more respectful, more peaceful, or more harmonious. Several curricula emphasized broader community-level changes, such as safer, more equitable communities. At the same time, programs for adolescents often highlighted individual benefits, such as improved confidence and leadership, as well as safer schools and communities.

This aspirational or positive framing was often established in the first session, where participants were generally introduced to the program and its goals. Two-thirds of curricula (62%, 34/55) used aspirational messaging, while the remaining (38%, 21/55) tended to emphasize the problems or issues that would be discussed in the sessions. Curricula varied in how explicitly they acknowledged a program’s aim to prevent violence or promote gender equality. Less than half (47%, 26/55) the curricula explicitly mentioned the goal of reducing violence upfront, and these were primarily from VAWG programs. Curricula from other programs generally emphasized their other primary goals, such as SRH, economic empowerment, or family and child wellbeing. Only 14.5% of curricula (8/55) spoke explicitly about promoting gender equality when describing program goals.

Avoiding direct discussion about violence or gender early in a program may be a deliberate strategy to reduce resistance, although program implementers did not always acknowledge it. One curriculum, however, described taking a “gender without the g word” approach: framing messaging around family and parenting rather than gender and masculinities, to reduce resistance when recruiting and working with men [22] (p. 27).

#### 3.2.2. Gender and Power Were Core Topics Within Curricula, Alongside Violence and Healthy Relationships

There was considerable commonality in the core subject matter of curricula, despite differences in program goals, entry points, and populations. Five topic areas emerged as the most common across curricula: violence, gender, healthy relationships, power, and communication, however they did not necessarily comprise the largest proportion of content in individual curricula. Each topic area was present in at least three-quarters of curricula (76%), and 54.5% (30/55) of curricula addressed all five topics. On average, a curriculum included four out of the five topic areas. Family strengthening, SRH, and economic strengthening program curricula were more likely to address all five topics compared to VAWG program curricula. The five core topic areas are described below.

##### Topic Area 1: Understanding and Addressing Violence

All but one curriculum (98%) included explicit content on violence, often focused on IPV, but also covering other forms of violence against women or girls or interpersonal violence. For example, curricula for boys and men commonly encouraged reflection on different forms of violence participants had experienced, witnessed, or perpetrated. The topic of violence was often introduced in later sessions, once rapport had been built with participants, particularly for SRH, economic, or family strengthening curricula. Content on violence encompassed three main subtopics: understanding violence (98% of curricula); taking action to prevent violence (67%); and supporting survivors (64%). Three-fifths of curricula (62%, 34/55) addressed all three subtopics, though violence made up a larger proportion of content in VAWG curricula than those from other programs.

The subtopic of understanding violence generally focused on defining violence and its different forms (e.g., physical, sexual, emotional/psychological, economic). Only four curricula (7%), all for adolescents, discussed technology-facilitated violence (e.g., cyber violence, online harassment, image-based abuse, using technology to monitor a partner). Curricula encouraged reflection on the key risk factors or triggers for violence (e.g., alcohol abuse, conflict, lack of communication, economic stress, jealousy) and their consequences. Some curricula emphasized that violence is a learned behavior and can therefore be unlearned. Curricula that were well adapted to local contexts used culturally relevant case studies, or local proverbs that normalize men’s use of violence, to spark conversation about the prevalence and acceptability of violence. A few curricula made connections between interpersonal violence and broader community or structural violence, including legacies of conflict.

Violence was commonly described as a ‘cycle’. In some curricula, this cycle referred to three phases of an abusive relationship: anger or tension building, erupting into violence, followed by a partner’s apologies and promises to stop (sometimes called ‘the honeymoon phase’) before the cycle repeats. However, the data underlying this theory, developed in the 1970s in the United States, and its generalizability have been critiqued for decades [23,24]. In other curricula, the cycle of violence referred to how exposure to violence increases the likelihood of experiencing or perpetrating it in the future, though this was not always clearly explained to participants. Despite its importance as a risk factor for IPV, only a few curricula encouraged deeper reflection on how childhood experiences of violence can shape future risk of violence.

The second subtopic, taking action to prevent violence, encouraged participants to identify key steps to prevent or interrupt violence, including raising awareness in their communities, being active bystanders who intervene when witnessing violence, and developing individual or group action plans. These activities also often focused on ways to foster more equal relationships between men and women, including sharing household responsibilities. Curricula designed to reach only men included a larger proportion of sessions on taking action compared to those for only women or for men and women.

The third subtopic, supporting survivors, sought to dispel shame and stigma for survivors of violence and encourage seeking help or support, which sometimes included the creation of individual safety plans. Group discussions supported participants to reflect on the reasons why survivors might not seek help and how participants could provide stigma-free support to (primarily) women experiencing violence. Curricula delivered to only women had a greater proportion of sessions on supporting survivors compared to those for only men or for men and women.

Less than a third of curricula (31%) also addressed violence against children (VAC), defining types of violence children experience (e.g., violent discipline by caregivers or child sexual abuse), consequences for children’s health and development, how to support children experiencing violence, or the intergenerational transmission of violence. VAC content was most common in programs for adolescents and in family strengthening curricula. Notably, all family strengthening curricula addressed both VAW and VAC, but were included because of their specific aim to prevent IPV and therefore may place greater emphasis on IPV than other family strengthening or parenting programs.

##### Topic Area 2: Questioning Gender Norms and Roles

Nearly all curricula (94.5%) included critical reflection on gender norms and roles, including all 14 curricula delivered with only men or boys. Many curricula addressing gender (75.5%) introduced the concept in the first or second session, despite many not being explicit about their aim of promoting gender equality. Activities and information sharing to differentiate ‘sex’ from ‘gender’ were common. Sessions often focused on dispelling misunderstandings (e.g., that gender refers only to women) and helping participants understand that gender is a social construct. Interactive exercises and group discussion were used to prompt reflection on societal expectations for how men and women (or boys and girls) should behave, where these norms are learned, and the costs or consequences for those who do not conform. Only a handful of curricula, mostly for young people, addressed the concept of gender identity.

The metaphor of the ‘gender box’, the pressure to fit within a narrowly defined set of gender roles based on biological sex, and activities such as ‘Act Like a Man/Act Like a Woman’ were widely used. Credited to the Oakland Men’s Project in the United States (1980s), these tools illustrate the constraints of rigid gender norms and encourage participants to imagine the benefits of living without them [25]. Curricula for men and boys often explored masculinity (or the ‘man box’) and the consequences of restrictive gender roles for men themselves.

Contextually adapted curricula often referenced local sayings or practices to illustrate the influence of gender norms in daily life. Some curricula asked participants to reflect on how men’s and women’s roles have changed since their parents’ or grandparents’ time, to illustrate that gender roles are not natural or fixed but change over time. Most curricula included activities to think about how participants can support each other in adopting more equitable behaviors or promote gender equality in their families and communities. Some curricula went further to discuss patriarchy, gender inequality, or discrimination against women and girls at a societal level.

Some curricula successfully integrated discussion of gender norms across multiple sessions and topic areas, highlighting how they influence men’s and women’s (or boys’ and girls’) different opportunities and expected behaviors. For example, a parenting session in a family strengthening curriculum might ask parents how societal expectations influence fathers’ and mothers’ involvement in caring for their children. The curriculum may go further in challenging prevailing gender roles by encouraging fathers’ active involvement in childcare and household tasks. Similarly, an SRH curriculum might encourage participants to reflect on gender stereotypes that imply that women are solely responsible for preventing pregnancy, while encouraging healthy couple communication and shared responsibility for contraception.

##### Topic Area 3: Fostering Healthy Relationships

The topic of healthy relationships was addressed in 85.5% of curricula, and often focused on healthy romantic or partner relations, but sometimes extended to peer, parent–child, and other relationships. Common activities asked participants to roleplay or discuss the characteristics of ‘healthy’ versus ‘unhealthy’ (or ‘happy’ and ‘unhappy’) relationships, and how they look and feel. Healthy relationships were described as respectful, trusting, supportive, and equal. They also required mutual understanding: to be able to see the other person’s perspective, have empathy, listen, and openly communicate.

Healthy partner relationships were characterized as caring and loving, and some curricula emphasized partners sharing responsibility for household tasks or decision-making. Some, but not all, curricula emphasized the importance of personal autonomy and agency, with partners having freedom to make their own choices. Many curricula discussed consent, but this often took place in sessions on sex and sexuality rather than healthy relationships.

Activities and group discussions often asked participants to consider what they can do to build healthier (or more loving) relationships, with an emphasis on improving communication, expressing appreciation, and spending positive time together. Several curricula included exercises or take-home assignments to explore ideas about love and intimacy, or to assess the quality of one’s relationship. A few curricula discussed the importance of setting boundaries in a relationship but often did not go into detail on how to establish or manage them.

Content on healthy relationships was generally more common in curricula implemented with women only or with men and women, compared to those implemented with men only. However, all eight curricula that did not directly address healthy relationships included content on both effective communication and conflict resolution, skills that support healthy relationships.

##### Topic Area 4: Recognizing and Balancing Power

A slightly smaller percentage of curricula (76%) addressed power dynamics, which mostly focused on recognizing power imbalances between men and women. Power served as a foundational concept, although it comprised a smaller proportion of sessions compared to other core topic areas. Curricula often referenced power and power imbalances when discussing violence, relationships, sex, and decision-making. Activities commonly supported participants to understand different types of power (e.g., power *over*, power *to*, power *with*, and power *within*), with considerable focus on how power is used over others.

These four types of power emerged from feminist, development, and sociological thinking in mostly high-income countries in the 20th century [26] and have become popular in violence prevention programs. SASA! developers in Uganda found that framing discussions around power made it easier to discuss inequalities between men and women and often provoked less backlash than the language of gender or rights [27]. That said, practitioners have found that these four types of power sometimes do not translate well or work in some contexts [26,28].

Curricula included group discussions to explore who has power, status, or privilege and who does not, and what it feels like to be powerful or powerless. Curricula usually asked participants to reflect on how power can be used in positive and negative ways, how men and women use or experience power differently, and the consequences of unbalanced power for individuals, families, and communities. Experiential exercises were common to help participants visualize and feel power differences between individuals and within society. ‘Persons and Things’ [29] (p. 59), a common activity, divided participants into ‘persons’ who can act and make decisions, and ‘things’ who must do what the persons wish, to experience what it feels like to hold or be denied power. In another widely used activity, ‘The New Planet’ [30] (p. 6), participants simulated living in a new civilization where rights are granted and then arbitrarily taken away by those with more power, to illustrate how power shapes everyday life and relationships.

Participants were often asked to identify steps they could take to balance power in their relationships and propose solutions to reduce broader inequalities in society. Some curricula made clear links between gender and power, illustrating how unequal gender roles and unbalanced power interact to foster conflict and violence, reduce agency and autonomy, and create unhealthy relationships. By doing so, these curricula enabled participants to consider the potential benefits of less rigid gender roles and relationships grounded in equality and shared power. Some curricula also explored how gender intersects with other forms of discrimination, such as race or caste. However, no curricula explored the role that colonialism played in establishing or codifying hierarchies of power based on gender, or the ways that capitalism reinforces them, which could be explored in many of the contexts where these programs operate.

##### Topic Area 5: Promoting Effective Communication

Three-quarters of curricula (76%) promoted effective communication, often highlighting the potential risks of poor communication for relationships and wellbeing. Some curricula used interactive exercises, such as passing a whispered secret or guiding a blindfolded partner, to demonstrate how poor communication can lead to misunderstanding or frustration. Group discussions explored how different communication styles can be threatening, humiliating, or manipulative. In contrast, effective or good communication was described as honest, open, clear, direct, respectful, and requiring empathy and self-awareness. Both verbal and nonverbal communication skills were addressed, and curricula often included activities aimed at building these skills. For example, having group members (or partners) practice active listening or communicate their thoughts and feelings clearly. Some curricula designed for couples extended this into homework assignments to promote couple communication.

Most curricula focused on promoting assertive communication (62%, 26/42), which emphasizes using ‘I’ statements to communicate thoughts, feelings, and emotions. Assertiveness was often contrasted with aggressive, passive, or (somewhat less commonly) passive-aggressive communication, or variations of these styles. Assertive communication is valued in individualist societies that prioritize direct expression, such as the United States, where assertiveness training came to prominence in the 20th century [31]. While highly adaptable, the relevance of promoting assertive communication has been questioned in collectivist societies, which value group harmony and more indirect and relational ways of communicating [32,33].

Communication activities were often paired with content on conflict resolution (addressed in 65% of curricula). Yet relatively few curricula addressed two common sources of conflict that require effective communication: household decision-making (33% of curricula) and managing household finances (22% of curricula). Economic strengthening curricula devoted a greater proportion of sessions to these topics than other programs.

#### 3.2.3. Relatively Few Curricula Directly Addressed Harmful Alcohol Use or Poor Mental Health, Despite Their Substantiated Links with IPV

Less than a quarter of curricula (24%) included content explicitly focused on managing or reducing alcohol consumption, despite alcohol use being a key risk factor for experiencing or perpetrating IPV [34]. The limited focus on alcohol is surprising given that most curricula acknowledged it as a risk factor or trigger for violence (and other risky behaviors). Existing content on managing alcohol was concentrated in a few programs (two programs accounted for six of the 13 curricula), and curricula drew on a relatively limited number of adapted activities. For example, five curricula adapted the activity ‘Throw the Drunk Ball’, which asks men to consider the reasons they drink and the links between alcohol and ideas about masculinity [35].

These curricula typically began by asking participants to reflect on the reasons people drink (or use drugs) and the potential risks or consequences for themselves and their families. One curriculum for young men described the signs and symptoms of alcohol dependency and encouraged young men to reflect on their relationship with alcohol. Activities to help participants manage or reduce their own (or a partner’s) alcohol consumption had participants brainstorm specific strategies or steps to take, share tips, and identify sources of support. A few curricula also encouraged participants to limit alcohol availability in their communities, for example, by organizing actions to protest the opening of new alcohol outlets.

Similarly, relatively few curricula (16%) included intensive or targeted efforts to address poor mental health, despite the evidence that it increases the risk of perpetrating or experiencing IPV [34]. That said, many curricula mentioned poor mental health as a consequence for women (or children) experiencing violence. Curricula that specifically addressed poor mental health were often developed for conflict-affected settings and were trauma-informed, designed with an understanding of how trauma affects participants’ health, behavior, and well-being.

Mental health content included discussion of mental health and illness (e.g., depression, self-harm, or suicide), trauma and how it affects the body, and how to recognize symptoms of poor mental health. These curricula devoted substantial time (e.g., entire sessions) to developing skills to cope with stress or trauma, such as mindfulness, deep breathing, or relaxation. A few curricula integrated strategies to help participants identify and address negative thought patterns and how these influence behaviors. Group discussions also focused on psychosocial needs, challenged stigma around mental health, and provided information on locally available mental health services to encourage help-seeking.

It is worth noting that many more curricula included some content on emotional regulation, stress, or brief relaxation activities that can support positive mental health. However, these were often short, not reinforced across sessions, nor accompanied by information on where to seek help. Notably, only two curricula explicitly addressed both alcohol and poor mental health, a gap given how commonly the two co-occur.

#### 3.2.4. Most Curricula Aimed to Strengthen Relationship Skills, but Many Included Relatively Limited Instruction or Time for Learning These Skills

Many curricula sought to build skills to support respectful and non-violent relationships, primarily communication (76%), conflict resolution (65%), and social and emotional (65%) skills. Sessions on conflict resolution usually focused on discussing common sources of conflict and how to defuse or resolve conflict respectfully and non-violently. This usually involved role playing scenarios of relationship conflicts and putting into practice new communication and/or emotional regulation skills.

Content on social and emotional skills included exercises to help participants identify common emotions (e.g., fear, anger, sadness, happiness, affection), discuss healthy expression of emotions, and build skills to regulate emotions like anger (more often in curricula for men). Some curricula included activities to help manage or cope with stress or to build self-esteem and confidence. Family strengthening curricula included a greater proportion of sessions on social and emotional skills compared to other programs.

Behavior change theories suggest effective skill-building includes: a clear description of the skill broken into smaller steps; correct demonstration of the skill; time to observe and critique the performance of the skill; practice to build self-efficacy; opportunity to receive feedback from peers or facilitators; and setting intentions to use the skill in daily life [36,37,38]. The curricula we reviewed were strong at fostering reflection and motivating participants to adopt new skills. For example, helping men understand the benefits of communicating their feelings and challenging attitudes that might discourage this, like the idea that men should be tough and not express emotions. Yet many curricula fell short in providing the level of instruction and practice time required for participants to learn these new relationship skills.

Curricula were not always clear on the specific skills or behavior they wanted participants to adopt, sometimes focusing more on what participants should not do (i.e., use violence), as opposed to what they should do instead. Step-by-step instruction and modeling of skills was limited. For example, participants were often asked to role play resolving conflicts after only a group discussion or brainstorm, but with no instruction or modelling of the relevant skills. Curricula often allocated limited time for participants to learn and practice skills, and it was unclear whether facilitators provided constructive feedback to help participants improve or develop self-efficacy in performing the skill.

In contrast, curricula that drew more closely on evidence-based practice devoted whole (or multiple) sessions to skills-building, included practical demonstrations and step-by-step instructions, allowed sufficient time for practice, and encouraged the application of new skills through homework assignments. Interestingly, some curricula provided more thorough instruction and time for learning other, more tangible skills, such as caring for a baby, than they did for relationship skills.

### 3.3. Findings on Curricula Teaching Approaches, Learning Methods, and Adaptation

This section examines the teaching approaches and learning methods employed by curriculum-based programs, followed by findings on program adaptation and the use of content from earlier programs.

#### 3.3.1. Curricula Were Grounded in Participatory Teaching Approaches and Prioritized Creating Conducive Learning Environments

Curricula used participatory teaching approaches that engaged group members as active participants in the learning process. Curricula were grounded in the pedagogy of Paulo Freire, who advocated for critical reflection and dialogue to raise individual and collective ‘critical consciousness’ (an awareness of social inequalities and their root causes) and collective action to challenge injustice [39]. Curricula primarily adopted Freire’s use of critical reflection and dialogue, asking open-ended, problem-posing questions that encourage participants to question existing beliefs and engage with new concepts such as gender and power [6,40]. Only a few curricula systematically promoted the collective action part of Freire’s approach.

In contrast to traditional, lecture-based teaching approaches, participatory approaches emphasize shared power between facilitators and participants. Many curricula reflected this by proposing a full or semicircle seating arrangement, in which facilitators sat alongside rather than in front of participants. Facilitators of the learning process were sometimes described as co-learners, although in practice they were asked to accurately convey information and answer participants’ questions.

Program implementers and curricula stressed the importance of establishing supportive environments to enable active learning. This included creating spaces where participants felt safe to interact and share experiences and opinions. Programs often began by allowing participants to get to know each other and understand what to expect in the program. This was usually (85% of curricula) followed by adopting ground rules (e.g., respect, listening, active participation, voluntary sharing) to promote comfort and trust and reduce potential shame or embarrassment. Conversations about confidentiality were particularly important to address participant fears that their personal stories would be disclosed to non-members.

#### 3.3.2. Curricula Used a Variety of Participatory Facilitation Methods to Prompt Discussion and Reflection and Build Skills

Curricula emphasized discussion and critical reflection to support attitudinal change, alongside experiential learning to adopt new skills and behaviors. A variety of participatory facilitation methods were employed to promote interaction and positive peer norms, and support participant learning by doing. Large-group discussion and brainstorming were the most common facilitation methods, alongside case study or scenario discussions, role-plays, small-group work, peer-to-peer or couple discussions, games, and experiential activities designed to build skills.

Metaphors were commonly used to help communicate complex or abstract concepts, like ‘gender’ (e.g., the gender box), ‘power’, or the root causes of violence. Homework assignments were also common and often encouraged participants to speak to a partner or friend about new concepts they had learned or to practice a new behavior. Participants were sometimes invited to share their homework experiences at the next session. Slightly less common were visioning or goal-setting exercises (more common in family strengthening curricula) or the development of individual, family, or group action plans.

Less common methods included oral quizzes, debates, competitions, drawing, and written exercises (e.g., letter writing, making a budget). Song and dance were encouraged as energizers but were limited in core curricular activities. Only seven curricula (13%) integrated audio-visual materials in group sessions, including videos (6) or radio dramas (1). A few programs integrated sports (e.g., rugby, cricket, football) within group sessions or as parallel activities. One program provided a phone-based application to aid facilitators in preparing sessions.

The use of schema, including mnemonics or acronyms, to help participants with memory, recall, and application of key information or practices was surprisingly limited. Examples to support effective couple communication included the CLEAR rules (Conversation, Listening, Encourage, Appreciate, Respect) [41] or the 4Es (Express No, Explain why, Explain what you want, Ease the tension) [42]. Some programs provided participants with workbooks or handouts for specific sessions. Notably, curricula did not always distill learning into key messages or reinforce and repeat them.

#### 3.3.3. Curricula and Their Content Were Often Derivative of Earlier Programs, but the Degree of Adaptation Varied Greatly

Adaptation of previous programs or their content was widespread. More than one-third (36%) of curricula were direct adaptations or iterations of an earlier program. Adapted curricula at times closely resembled their source materials, while others were significantly adapted for a new context, with a stronger focus on violence prevention, and/or to integrate new program aims (e.g., SRH). The developers of the original sources were sometimes, but not always, involved in their adaptation.

Another quarter of curricula (25.5%) were described by program implementers as ‘inspired’ by or ‘infused’ with the approach of an earlier program. Some curricula merely adopted the facilitation methods of the earlier program, but not much content. With a few exceptions, there was relatively limited information describing how or why a particular curriculum was selected for adaptation, or the process undertaken to adapt it to the new context and/or populations (e.g., formative research, community consultation, co-development, pre-testing or piloting).

All but five curricula (91%) included content adapted from earlier manuals or curricula, such as participatory activities, guided group discussion, or core concepts. Adapted content often reflected the five core topic areas as well as the gendered distribution of unpaid care work, sexuality and consent, and SRH. Activities were often lightly modified, but at times were almost unrecognizable. Curricula in the review cited content adapted from 93 distinct manuals, curricula, or toolkits. These materials were mostly developed to support gender transformative or SRH programming, primarily in LMICs (84%, 78/93). On average, a curriculum cited activities or content adapted from four sources, and as many as 11. This includes content (activities or information) adapted from earlier programs (i.e., manuals, curricula, toolkits). Many curricula also cited information or definitions, drawn from other sources such as public health guidelines or GBV laws and policies, which were not counted. A few curricula included recognizable content but did not cite any source.

A large portion of adapted content was drawn from five resources [29,43,44,45,46,47] developed to promote gender equality and advance HIV and/or violence prevention. Some curricula drew considerable content from these sources, while others adapted an activity or two. Four of the five resources were made openly available specifically to allow organizations to contextually adapt them, which may explain why their content was so commonly adapted. However, a small number of individuals and organizations were responsible for developing several curricula in the review, which also explains some similarity in content.

The five curricula that did not cite any adapted content addressed many of the same topic areas as other curricula [41,48,49,50,51]. These curricula were described as developed through intensive formative research with communities, human-centered design processes, or relied extensively on existing organizational knowledge and approaches [40,52,53,54]. One of them, Sisters for Life [49], is among the oldest in the review, and seven other curricula cited content adapted from it. More details on adaptation can be found in Supplemental Table S5.

#### 3.3.4. Curricula Were Not Always Grounded in Theory and Content Was Not Always Connected to a Theory of Change

Programs cited several social and behavioral theories that informed program or curricula design. Most prominently this included social cognitive theory, the theory of gender and power, social norms theory, Freire’s conscientization, social ecological theory, stages of change theory, and the theory of planned behavior. However, only a few programs detailed how theory informed the program design or curricula content, which was most often found in program documents or articles describing program development.

Yet, there was sometimes a weak relationship between these underlying theories and curricula content and learning methods. For example, some curricula that cited social cognitive theory did not include any practice, rehearsal, or observation of new behaviors, a key element of the theory [36]. The weak theoretical basis for some curricula may to some degree reflect the widespread adaptation of programs and content, with theories behind the original programs being cited without critical engagement with them.

Further, curricula content and methods were not always clearly linked to a theory of change (ToC), which should articulate how a program is expected to operate and why it is expected to produce change based on the context, population, and local risk factors [4,55]. ToCs were not available for all programs or curricula. Where available, some ToCs were not specific to the curriculum-based component but described how the interactions between multiple components of a broader program were expected to produce change.

Only a small proportion of available ToCs clearly described the targeted attitudes or behaviors (e.g., improve emotional regulation and communications skills) and pathways or mechanisms through which these changes would reduce violence (e.g., men and women communicate and negotiate effectively). Further, curricula content did not always or adequately address the specific attitudes or behaviors outlined within the ToCs, or vice versa.

### 3.4. Findings on Curricula Implementation and Facilitation

This section presents findings on program implementation (summarized in Table 3) and program facilitators and their training. The review sought to clarify implementation details often missing from published descriptions, to better understand how curriculum-based programs are delivered. However, data were missing or limited for some implementation features, particularly implementation quality or fidelity, which we do not discuss.

#### 3.4.1. Session Number, Frequency, and Instruction Time Varied Considerably, but a Sizeable Proportion of Curricula Were of Limited Duration

We found considerable variation in program design and curricula implementation. While the number of sessions was commonly used to describe curriculum-based programs, this obscured considerable variation in instruction time, session frequency, and program duration. Total instruction time ranged from six to 96 h and was 34 h on average, although 56% of curricula were shorter. Depending on the number and frequency of sessions, participants were engaged in direct programming from two days up to three years.

Curricula comprised between six and 60 sessions (16 on average), but some programs delivered multiple sessions in a single meeting. Most curricula (60%) were delivered on a weekly basis, though some programs tapered session frequency over time (e.g., shifting from weekly to monthly meetings). This happened by design or was a response to the limited availability of participants or facilitators for some programs.

Evidence and practice-based knowledge suggest programs need to be long and intensive enough to achieve change, allowing time between sessions for participants to internalize and apply new attitudes and behaviors [8,56,57]. A recent review of the What Works to Prevent VAWG program found that successful programs ran between 40 and 50 h [4,8]. Only nine curricula (16%) in this review were within that range, and a sizeable proportion (36%) were less than half as long. Several curricula were delivered within a relatively short period, including through one-off workshops, leaving little time for participants to learn and apply new attitudes and behavior in lasting ways.

#### 3.4.2. Participant Recruitment and Attendance Were Common Challenges for Which Programs Adopted Different Strategies

While participant eligibility criteria were generally clear, we found relatively limited information detailing recruitment processes or messaging. Program implementers cited recruiting participants and keeping them engaged as common challenges. Some programs recruited through existing community groups or institutions, to help identify individuals meeting specific criteria or living stably in the community. Some curricula were also delivered directly within these spaces, which allowed programs to reach people where they were and potentially bolster attendance. More than a third (34.5%, 19/55) of curricula recruited through existing groups or institutions, such as savings groups, schools, workplaces, youth clubs, sports groups, self-help groups, and places of worship. This included half the economic strengthening curricula (50%) and nearly half the VAWG curricula (44%).

Programs used different strategies to encourage regular attendance and overcome common participation barriers. One strategy was to hold meetings at accessible locations and convenient times for participants, which were sometimes identified through formative research with communities. Some programs allowed group members to decide on the day and time for the meetings, and less commonly, the meeting place. A few programs were implemented in participants’ schools or workplaces, but they were not immune to challenges, with time constraints sometimes hindering the regular delivery of sessions.

Beyond scheduling, some programs offered financial compensation, material or in-kind support, or other tokens of appreciation designed to variously motivate attendance, remove barriers to participation, or acknowledge people’s time and investment. Of the 26 curricula that reported this information, about half (52%) reported providing some form of support or compensation. Material supports included basic household or food items, sports equipment, or branded gifts (e.g., hats, bags, t-shirts). Financial support was usually cash to pay for transportation or compensate potential income loss during sessions. Refreshments including beverages, snacks, or lunch were provided in some programs, which acknowledged the difficulty of learning on an empty stomach. Five of the eleven curricula that reported they did not provide any type of support or compensation were part of economic strengthening programs. Participants or their family members in these programs may have received other benefits through their participation in savings groups, income generation or training opportunities.

Despite these strategies, some programs found the planned timing, duration, or frequency of sessions were at odds with facilitator or participant availability, especially for male participants. As a result, some programs had to shorten sessions and/or reduce their frequency, diluting program content and connection with participants. However, programs used very different metrics to report attendance data, and such information was not reported for 40% of curricula. Overall, we found that 42% of programs with data reported participants attended less than 70% of sessions, a considerable challenge for achieving and sustaining behavior change.

#### 3.4.3. Large Group Sizes for Some Programs May Have Hindered Their Aims for Participatory Learning and Interaction

Curricula were delivered in groups that included between six and 50 members. Many programs recommended (or reported) a minimum and maximum number of participants, but some programs provided strict guidance or relied on the membership of an existing group. Groups included between 17 and 23 members on average, based on available data. Larger than average group sizes were seen in economic strengthening programs and programs with adolescents; often reflective of their delivery in schools or existing youth or savings groups.

Group size also affected facilitator workload. Where a single facilitator was used, groups averaged between 12 and 17 participants. Where two or more facilitators were paired together, the number of participants per facilitator was better (1:11 vs. 1:16). Pairing facilitators together allowed groups to be divided for smaller group discussions and targeted instruction and also enabled facilitators to provide individual support to those who might need it without disrupting the session, while also sharing the workload.

Some of the larger group sizes seen in the review are at odds with the participatory, peer-based learning approach underpinning these programs. Smaller group sizes can be more conducive to participatory learning, make participants feel comfortable to voice their ideas, and enable them to develop supportive relationships [58]. Yet, some programs had as many as 40 to 50 participants, and some programs invited participants’ partners and/or children to certain sessions, requiring facilitators to manage a group double or triple in size.

#### 3.4.4. Most Programs Delivered Curricula to Both Men and Women, Often Separately, and Sometimes with Different Content

Most curricula (82%) were delivered within programs that took a gender synchronized approach (45/55 curricula), meaning they worked in intentional and coordinated ways with both men and women (or boys and girls) to challenge gender norms [59]. These programs worked with both sexes, who were sometimes peers, couples, or of different ages, separately or together. Nearly a third of these curricula (31%) were implemented in mixed-sex groups.

Many more gender synchronized curricula were delivered, fully or partially, to men and women separately. Programs implemented separate men’s and women’s groups (40%), usually concurrently, or included a combination of both single- and mixed-sex sessions (29%). The latter included programs that brought separate men’s and women’s groups together periodically to discuss specific topics or combined them part-way through implementation, as well as curricula that worked primarily with one partner (often men) but invited the other partner to specific sessions.

Program implementers highlighted different, often context-specific reasons for whether to hold separate or joint sessions with men and women. Programs that delivered curricula in single-sex groups (or a combination of single- and mixed-sex groups) often emphasized the importance of ensuring participant comfort and predominantly women’s safety. They acknowledged individuals might not feel comfortable speaking about sensitive topics in the presence of the opposite sex, including their partners. Programs that included all (or some) mixed sessions often emphasized the importance of creating space to foster dialogue between men and women (or partners). However, some of these curricula also divided participants into separate, single-sex groups within sessions when discussing sensitive topics like sexual health or violence.

Gender synchronized programs sometimes provided different content to each sex, including in more than half (60%) the programs reaching couples. These differences in content were sometimes surprising and might undermine program goals. For example, the women’s curriculum in one program discussed the importance of couples balancing power and sharing decision-making. Yet the men’s curriculum did not address these topics, but rather focused on engaging men in household tasks and childcare. Encouraging women to seek more balanced power in their relationships may be difficult, and potentially harmful, if their partners have not been exposed to similar content and ideas.

Notably, only ten curricula (of 55) came from programs that engaged only one sex in curriculum-based programming to challenge unequal gender and power dynamics. Six of these ten programs included the opposite sex in other program components, such as economic empowerment interventions or community mobilization efforts. These ten programs all began implementation prior to 2015, by which time there had been calls from some prevention researchers and advocates to ensure programs engaged both women and men [59,60].

#### 3.4.5. Facilitators Were Critical to Program Success, Came from Diverse Backgrounds, and Were Often the Same Sex as Participants

Program implementers and researchers consistently emphasized how crucial facilitators were to program success. Effective facilitators were described as capable to build mutual understanding and respect, encourage participants to question long-held beliefs, maintain cohesion among group members, and handle complex and sensitive topics with care. Programs generally sought individuals with characteristics and skills that could support these processes, including being open-minded, non-judgmental, empathetic, active listeners, good communicators, supportive of gender equality, and motivated to support others. Several curricula suggested that facilitators should be humble and self-aware, and not afraid to share their own experiences or acknowledge that they were also ‘works in progress.’

Most curricula (91%) were facilitated by NGO staff or field agents (40%), community leaders (27%), or peers (24%), as shown in Table 4. Community leaders were generally respected community members with key characteristics sought by program implementers and sometimes included elected or faith leaders. Peer facilitators were intentionally similar in age and background to group members, for example, from the same community or with similar life experiences. They were sometimes selected or elected by participants, from within the group or the broader community. Several programs engaged external individuals with specific expertise (e.g., health care providers, counsellors) to co-facilitate sessions on health, violence, or local laws and services.

Facilitators were often the same sex as group members (86%, 42/49), which program implementers noted could foster trust and help participants feel comfortable and safe. Slightly less than a third of curricula (15/55) intentionally utilized both male and female facilitators. One curriculum had male and female facilitators lead separate single-sex groups. Whereas 14 of these curricula paired male and female facilitators together to co-facilitate sessions, either exclusively with mixed-sex groups (5) or when bringing separate men’s and women’s groups together for specific sessions (9). Program implementers reported that having a male and a female co-facilitate provided an opportunity to model more equitable relationship dynamics within the group and allowed facilitators to conduct separate, single-sex discussions on sensitive topics when needed.

While the general characteristics or backgrounds of facilitators were often documented, the process for identifying and recruiting them was rarely described in detail, suggesting greater documentation would be valuable.

#### 3.4.6. Facilitators Received Vastly Different Amounts of Training Despite Their Crucial Role, and Training Details Were Often Sparse

Despite their crucial role, facilitators received vastly different amounts of training. Facilitator training often combined reflection on facilitators’ own attitudes about gender and violence with practice-based learning on how to facilitate the curriculum. Training was conducted prior to and sometimes during implementation, which included training facilitators on specific sessions or modules, timed to coincide with their implementation, or refresher training. Several programs had facilitators first experience the full curriculum as participants, including as part of curriculum pre-testing, before training them to deliver it to others. However, information was often limited to brief descriptions of training duration or approach, with little detail on the content, structure, or persons who delivered it.

Research and practice-based knowledge indicate facilitator training needs to be of sufficient duration to provide instruction on curriculum subject matter, build participatory facilitation skills and confidence, and provide space for personal reflection on facilitators’ own attitudes about gender and violence [4,56,61,62], which have been associated with program reductions in IPV [63]. Facilitator training ranged from 3 to 30 days for the reviewed curricula (10 days on average), but duration was not stated or unclear for 31% of curricula. Only three-fifths of curricula (with data available) provided at least ten days of training, which is generally recommended for facilitators of gender-transformative programs [4,61,62]. Sufficient training is particularly important for those with no prior facilitation experience and/or knowledge of gender. Yet we found peer and community leaders received less training on average than NGO staff and field agents, although data were limited.

Most curricula (64%) included some guidance on facilitator roles and responsibilities, sometimes in an accompanying document. However, relatively few addressed how to manage difficult interactions. Exceptions included curricula that provided clear guidance on dealing with backlash, which gave concrete examples of resistance such as denial or victim-blaming, explained why a participant might resist changing socially or culturally rooted behaviors, and offered step-by-step responses. Similarly, only a few curricula addressed handling disclosures of violence or the process for referring survivors to services.

Many programs mentioned providing supportive supervision or mentoring facilitators, but with little detail on what this involved. A few programs reported conducting session observations and providing facilitators feedback, holding mentoring sessions, or having regular check-ins with facilitators through calls or text. More information on training and supervision would help in understanding program outcomes and support replication.

## 4. Discussion

This review analyzed the design, content, and implementation of 55 curricula to provide a snapshot of curriculum-based IPV prevention programming in LMICs. It is unique in the number of programs assessed and in its comparison of curricula content and implementation features, rather than outcomes. The curricula reviewed represent a rich and varied body of work, which span diverse entry-points, contexts, and populations, and reflect the considerable time, dedication, and collective effort of individuals and organizations invested in this field.

Despite differences in aims, populations, and locations, curricula employed similar methods and core content, primarily addressing violence, gender, healthy relationships, power, and communication. Program adaptation was widespread: only five of the 55 curricula were not directly adapted from, or did not include content from, an existing program. This demonstrates organizations are actively learning from and building on existing approaches, and points to the value of open-source materials, from which much content was adapted. At the same time, it reveals an outsized influence of a small number of organizations and program models in shaping current practice, raising questions about whether we are doing enough to innovate as a field.

The findings highlight numerous opportunities to strengthen the design, content, and methodologies of IPV prevention curricula. Relationship skills-building is one crucial area. While most curricula aimed to build skills such as communication, emotional regulation, and conflict resolution, they often emphasized critical reflection and discussion over actual skills development. Program effectiveness may be improved by focusing less on awareness-raising and more on using evidence-based approaches to equip participants with healthy relationship skills, which includes adequate time for skill development and practice [64]. Although program outcomes were not a focus of this review, it is notable that one curriculum, which did not directly address violence, was nonetheless shown to reduce IPV [63]. One quarter of its sessions focused on enhancing couple communication skills.

Integrating content on harmful alcohol use and poor mental health is another important area for strengthening and innovation. Relatively few curricula addressed these topics despite being known risk factors for experiencing or perpetrating IPV [34]. Even with limited content, several programs in the review achieved reductions in alcohol consumption and/or improvements in mental health; see, for example, [65,66]. Evidence also indicates that IPV prevention programs that target alcohol or substance abuse achieve greater reductions in violence [7]. There are clear entry-points to integrate alcohol and mental health more intentionally, building on existing references to these topics within curricula, which could strengthen program effectiveness [67].

There is also room to update and innovate curricula core content. Some curricula relied on outdated concepts that no longer align with current evidence. One example is the ‘cycle of violence’, a three-step pattern of abuse theorized to produce the learned helplessness underlying ‘battered woman syndrome’. Developed in the United States, this theory has faced major critiques, both for assuming all violence follows the same pattern and for the quality of the data underlying that assumption [23,24]. Another area for innovation is around how foundational concepts, such as gender, power, or communication, are framed and taught in curricula. Much of this content is drawn from theories or approaches developed by individuals and organizations from high-income countries, and it does not always resonate with participants in the diverse cultural contexts where programs are implemented.

One way to innovate is by co-designing programs with local communities and drawing more on local and Indigenous knowledge [68,69]. For example, organizations and activists already discuss Indigenous femininities and masculinities, colonial legacies of violence, and how colonial regimes introduced or accentuated power hierarchies between men and women [6,68,69]. This knowledge could be harnessed more within curricula and may even help counter anti-gender backlash, and its claims that gender equality undermines traditional cultural values [70]. Critical reflection can also be expanded to specifically address other persistent beliefs linked to IPV, including notions of respect for male authority, male headship, men’s perceived entitlement to sex, and family privacy. IPV prevention programs often find these widely held beliefs difficult to shift, but curricula rarely deconstruct them explicitly [71,72,73].

There are also opportunities for IPV prevention programs to more intentionally address violence against children, which often occurs alongside IPV and shares common risk factors and social norms [74]. Recent evidence shows that programs can simultaneously reduce both IPV and violent discipline of children [74]. This is important because childhood exposure to violence is a significant risk factor for perpetrating or experiencing IPV in adolescence and adulthood [74]. Parenthood can be a powerful motivator for change and an opportunity to promote respectful relationships, particularly for men [54,75]. In fact, evidence indicates that IPV prevention programs that integrate parenting content achieve greater reductions in IPV [7]. Clear opportunities to integrate and strengthen VAC prevention in IPV prevention curricula already exist, as nearly half the curricula in this review worked with couples (45.5%) and a third addressed VAC or parenting.

This review also uniquely expands our knowledge of program implementation. Critically, we found a shift towards gender synchronized programs over the last decade. This approach recognizes that both women and men are involved in shaping and reinforcing unequal gender norms and power dynamics and therefore must be engaged in challenging them [59,60]. There is also some evidence that programs are more effective when they work with both men and women, rather than only women or only men [8,76]. However, our review also found considerable variation in program implementation, including intensity, frequency, group size, and facilitator training, components that contribute to program effectiveness [4,7,17].

The design and implementation of a sizeable proportion of curricula diverged from good practice currently promoted by the field, though many were developed earlier. Relatively few curricula were guided by a clear and contextually grounded theory of change, which detailed the risk factors targeted and mechanisms of change. Several curricula lacked coherence, with content that did not suitably match the program’s stated aims. Further, program duration and facilitator training fell below recommended levels for a considerable number of curricula. These findings underscore the need to strengthen prevention practice to support more effective programming, particularly given resource constraints and growing evidence that poor adaptation or implementation limit effectiveness and may cause harm [77,78,79,80].

Alongside these gaps in practice, there is a need for better documentation of program development and implementation. The difficulty we had obtaining basic information on implementation is notable. Reporting was particularly sparse on participant recruitment, on facilitator selection, training, and support, and on implementation quality and fidelity. This partly reflects a broader tendency to publish on program impact rather than on implementation, a gap compounded by journal word limits that leave little room to adequately describe program design or delivery [81,82]. Yet these often-missing details are critical for enabling effective adaptation and replication.

This review has several limitations. We were limited to curricula, evaluations, reports, and other documentation available in English, French, or Spanish. Despite a deliberate effort to include curricula implemented by smaller non-governmental or community-based organizations, some were likely missed. The curriculum versions we had access to were not always the final versions used by facilitators and may not fully reflect actual implementation. While we were able to clarify aspects of program implementation with some organizations, we could not systematically have implementers validate findings for each curriculum. Lastly, the review looks at curricula content and implementation at a specific moment in time, which may not reflect how these programs have since evolved.

Based on our findings, we offer six recommendations to strengthen the design, content, and implementation of curriculum-based IPV prevention programs. These recommendations require action across the field, from donors to implementing organizations, practitioners, scaffolding organizations, INGOs, researchers, and journal editors. We believe applying these recommendations can strengthen prevention practice and enhance the effectiveness of the next generation of IPV prevention curricula.

**Recommendation 1. Balance innovation in new program models, content, and methodologies, with intentional adaptation of existing ones**

- Develop and pilot ***new*** curriculum-based programs and content across different contexts and populations, including with under-represented groups
- Ensure content and methodologies are continuously updated to align with current evidence and remove outdated content
- Explore new entry-points and framings to address core concepts like gender and power, building upon local and Indigenous knowledge
- Develop new critical reflection activities that interrogate other beliefs and norms driving violence, such as male headship, respect for male authority in the family, a husband’s perceived entitlement to sex, and family privacy
- Experiment with media and technological solutions to enhance learning, strengthen delivery, and facilitate scale
- Use digital platforms and short videos to standardize content, test digital delivery to complement or reinforce in-person sessions, and develop digital and AI applications to provide facilitators with ongoing support as programs scale
- Further test and evaluate programs to clarify their essential components, illuminate scope for innovation, and better understand which content and models work in different contexts
- Build organizational capacity to select, contextually adapt, and iterate proven models, while maintaining their core components [83,84]

**Recommendation 2. Ensure greater connection and coherence between curricula pedagogy, content, and a well-articulated theory of change (ToC)**

- Ensure organizations devote sufficient time and resources to develop well-defined ToCs that addresses local risk factors for IPV and are clear about how change is expected to occur
- TOCs should draw from theory and existing evidence and practice-based knowledge about what contributes to violence reduction and should be co-designed with communities to ensure they are grounded in local realities and perceptions of violence [69,80,85]
- Ensure there are clear links between a curriculum’s ToC and its core content and methodologies, as well as the indicators and outcomes used to monitor and evaluate program effectiveness, including to test hypothesized mechanisms of change [7]

**Recommendation 3. Strengthen focus on relationships skills in curricula and apply evidence-based behavior change approaches**

- Organizations should be clear about which skills they aim to build based on their ToC, and ensure appropriate content and methodologies are reflected in curricula
- Increase focus on building culturally grounded communication and emotional regulation skills, known protective factors and mechanisms of change in IPV prevention programs [73,80,86]
- Apply evidence-based behavior change approaches, ensuring adequate instruction, modelling, and practice for participants to acquire skills and develop self-efficacy [36,37,38]
- Expand the strategies used to teach social and emotional skills, integrating evidence-based approaches from the education and mental health fields
- Evaluate program impacts on skills acquisition and assess the relative contribution of different skills and approaches to violence outcomes

**Recommendation 4: Integrate strategies to reduce alcohol use and improve mental health within curricula**

- Strengthen practitioner knowledge on how alcohol use and mental health shape risks of IPV perpetration and experience
- Adapt evidence-based and Indigenous approaches for managing alcohol or improving mental health for integration in IPV prevention curricula, including through collaboration with the alcohol reduction and mental health fields [34]
- Evaluate whether integration of these approaches leads to reductions in alcohol consumption, improved mental health, and greater reductions in violence

**Recommendation 5: Encourage more IPV prevention programs to address violence against children, thereby disrupting intergenerational cycles of abuse and supporting long-term IPV prevention**

- Strengthen practitioner knowledge on the co-occurrence of IPV and violence against children and their shared risk factors and social norms
- Integrate content on parenting and violence against children within IPV prevention curricula, where it makes sense based on populations and program aims
- Encourage reflection on the links between violence in childhood and adulthood within curricula, regardless of the population
- Collaborate with and learn from the parenting field, to strengthen coordinated violence prevention for women and children

**Recommendation 6: Place greater emphasis on implementation design, planning, quality, and documentation**

- Continue generating and disseminating good practice to guide program design, planning, and implementation, based on emerging evidence and practice-based knowledge
- Assess the barriers organizations face in applying good practice, such as knowledge, capacity, funding, or timelines, and advance potential solutions
- Encourage organizations to publicly document the steps taken to develop or adapt programs, and to make curricula openly available
- Develop minimum standards for reporting on program design and implementation, which include facilitator training and support
- Call on journal editors to require detailed descriptions of program design and implementation as appendices to research articles [81]
- Encourage greater use of implementation science to examine implementation quality and fidelity and document this learning [55,82]

## 5. Conclusions

This review provides the most comprehensive assessment of curriculum-based IPV prevention programs and their implementation in the LMICs to date. The findings highlight several areas for strengthening and innovation, which informed six recommendations to guide the design, content, and implementation of future curriculum-based programs. These recommendations are intended for the field at large, though we acknowledge some of them will require building donor understanding and advocating for changes in funding practices. We hope these recommendations will not only deepen our understanding of how these programs work but ultimately contribute to their effectiveness in preventing violence against women and girls.

## Figures and Tables

**Table 1:** Characteristics of included curricula.

Characteristics	Number	Percentage
**Region**		
East Asia and the Pacific	6	11%
Europe and Central Asia	2	4%
Latin America and the Caribbean	3	5%
Middle East and North Africa	1	2%
South Asia	15	27%
Sub Saharan Africa	28	51%
**Implementation Context**		
Conflict-affected or humanitarian ^a^	7	12.7%
Rural	22	40.0%
Rural and urban	5	9.1%
Urban or peri-urban	21	38.2%
**Program type**		
Violence against women and girls (VAWG)	25	45%
Sexual and reproductive health (SRH)	13	24%
Economic strengthening	10	18%
Family strengthening	7	13%
**Program implementers/developers**		
National or local NGO(s) or university(s)	10	26.2%
International or regional NGO(s)	13	38.1%
Partnership between international and national NGOs or universities	32	35.7%
**Population curricula designed for**		
Men and women (or boys and girls)	26	47.3%
Men or boys only	15	25.5%
Women or girls only	14	27.3%
**Age of participants receiving curriculum**		
Adults only (aged above 18 years)	43	78%
Adolescents only (11 to 18 years)	5	9%
Adolescents and adults	7	13%

^a^ Based on descriptions by program implementers or evaluators at the time of implementation.

**Table 2:** Common topic areas in IPV prevention curricula.

Topic Area	Curricula Including the Topic Area			All Curricula
	Number of Curricula with the Topic (%)	Proportion of Sessions with the Topic, Range ^a^	Average Proportion of Sessions with the Topic	Average Proportion of Sessions with the Topic
Understanding and addressing violence	54 (98.2%)	4.8–62.5%	29.4%	28.9%
* Subtopic 1: Understanding violence*	*54 (98.2%)*	*4.3–50.0%*	*19.8%*	*19.4%*
* Subtopic 2: Taking action*	*37 (67.3%)*	*4.5–31.3%*	*13.7%*	*9.2%*
* Subtopic 3: Supporting survivors*	*35 (63.6%)*	*4.3–37.5%*	*12.9%*	*8.2%*
Gender norms and roles	52 (94.5%)	6.7–33.3%	16.13	15.4%
Healthy relationships	47 (85.5%)	3.4–40.0%	15.4%	13.1%
Recognizing and balancing power	42 (76.4%)	1.7–16.7%	8.9%	6.8%
Effective communication	42 (76.4%)	4.2–55.6%	18.6%	14.2%
Resolving conflict	36 (65.5%)	2.5–25.0%	9.0%	5.9%
Social and emotional skills	36 (65.5%)	4.0–20.0%	10.3%	6.8%
Division of unpaid care work	35 (63.6%)	2.5–22.2%	9.3%	5.9%
Sexuality and consent	32 (58.2%)	5.0–50.0%	12.4%	7.2%
Sexual and reproductive health	30 (54.5%)	2.4–50.0%	17.3%	9.4%
Laws, rights and policies	22 (40.0%)	4.0–27.3%	11.0%	4.4%
Parenting and child development	20 (36.4%)	4.3–39.1%	21.6%	7.9%
Making (household) decisions	18 (32.7%)	4.3–50.0%	11.5%	3.8%
Violence against children	17 (30.9%)	6.3–33.3%	14.3%	4.4%
Managing alcohol & substance use	13 (23.6%)	2.5–50.0%	9.8%	2.3%
Managing household finances	12 (21.8%)	3.4–37.5%	12.3%	2.7%
Mental health	9 (16.4%)	4.5–18.2%	10.2%	1.7%
Health (other) ^b^	9 (16.4%)	2.4–20.0%	5.9%	1.0%

Italics denote sub-topics. ^a^ Number of sessions on the topic area divided by the total number of sessions for each curriculum; range displays the minimum and maximum observed. ^b^ For example, maternal health or men’s health.

**Table 3:** Curricula implementation characteristics (n = 55).

Characteristics	Number	%	Characteristics	Number	%
**Hours of instruction ^a^**			**Gender synchronized approach**		
Average hours	33.8	-	Both sexes engaged in curricula	45	81.8%
<10 h	1	1.8%	One sex engaged in curriculum	10	18.2%
10 to 17 h	12	21.8%	*Female participants only*	*5*	
18 to 24 h	15	27.3%	*Male participants only*	*5*	
25 to 39 h	7	12.7%			
40 to 50 h	9	16.4%	**Synchronized approach: sessions**	***n = 45***	
>50 h	10	18.2%	Separate, single-sex sessions	18	40.0%
Unclear	1	1.8%	Mixed-sex sessions	14	31.1%
			Combined single + mixed sessions	13	28.9%
**Total contact points** ^b^					
Average number	15.3	-	**Group size ^d^**		
<10	14	25.5%	Minimum members, average	16.8	-
10 to 15	19	34.5%	Maximum members, average	23.1	-
16 to 20	10	18.2%	Unknown/not reported	5	9.1%
21 to 30	9	16.4%			
>30	3	5.5%	**Number of facilitators ^e^**		
			1 facilitator	22	40.0%
**Meeting duration**			2+ facilitators	15	27.3%
Average (hours)	2.1	-	Unknown/not reported	18	32.7%
<1 h	2	3.6%			
1–2 h	24	43.6%	**Participant compensation or support**		
2–3 h	26	47.3%	Unclear/not reported	29	52.7%
>3 h	3	5.5%	None provided	11	20.0%
			Support provided ^f^	15	27.3%
**Meeting location**			*Material support*	*7*	*12.7%*
Community	46	83.6%	*Financial support*	*6*	*10.9%*
School	3	5.5%	*Food/drinks*	*3*	*5.5%*
Workplace	3	5.5%			
Place of worship	1	1.8%	**Participant attendance rates ^g^**		
Community + home	2	3.6%	Not available/reported	22	40.0%
			Low attendance ^h^	3	5.5%
**Meeting frequency** ^c^			Moderate attendance ^i^	11	20.0%
Twice weekly	6	10.9%	High attendance ^j^	19	34.5%
Once weekly	32	58.2%			
Every two weeks	8	14.5%			
Once monthly	5	9.1%			
Intensive workshop	2	3.6%			
Unknown/unclear	2	3.6%			

Bold denotes headings; italics denote subheadings. ^a^ Total hours of instruction for the primary program participant; some programs invited participants’ partners or other family members to specific sessions. ^b^ Total number of contact points with participants sometimes differed from the number of sessions in a curriculum (i.e., multiple sessions delivered in a single meeting). ^c^ Based on the frequency most reported; some programs varied or tapered session implementation (e.g., from once to twice per week). ^d^ Group size often reported as a range (e.g., 10–15); we report the average of the minimum and maximum group sizes reported. ^e^ Based on the number of facilitators leading the core sessions; some programs invited guests or combined groups for specific sessions. ^f^ Some programs gave more than one type of incentive; hence percentages do not add up to 100%. ^g^ Different metrics were used to report attendance rates and were converted for analysis. ^h^ Low attendance: primary participants attended less than 50% of sessions on average. ^i^ Moderate attendance: participants attended 50% to 70% of sessions on average. ^j^ High attendance: participants attended more than 70% of sessions on average.

**Table 4:** Facilitator characteristics (n = 55).

Characteristics	Number	Percentage
**Facilitator background**		
NGO staff or field agents ^a^	22	40.0%
Community leaders ^b^	15	27.3%
Peers ^c^	13	23.6%
Service providers ^d^	1	1.8%
Teachers ^e^	4	7.3%
**Facilitator sex**		
Unknown/not reported	6	10.9%
Male only	16	29.1%
Female only	12	21.8%
Male or female	6	10.9%
Male and female	15	27.3%
*Lead separate, single-sex groups*	*1*	*1.9%*
*Co-facilitate mixed-sex groups + lead single-sex groups*	*9*	*16.4%*
*Co-facilitate only mixed-sex groups*	*5*	*9.1%*
**Length of facilitator training**		
<5 days	6	15.8%
5 to 10 days	20	52.6%
11 to 15 days	10	26.3%
>16 days	2	5.3%
Unknown or unclear	17	30.9%

Bold denotes headings; italics denote sub-headings. ^a^ NGO staff or field agents: e.g., gender specialists or individuals with facilitation experience hired to deliver sessions. ^b^ Peers: individuals of similar background characteristics as participants. ^c^ Community leaders: respected community members, faith leaders, or elected leaders. ^d^ Teachers: delivering programs in the classroom or in the community. ^e^ Service providers: including health or other frontline workers.

## Data Availability

The data extracted and analyzed for this scoping review are available from the corresponding author upon reasonable request. Some of the curricula reviewed are subject to third-party restrictions and cannot be shared publicly.

## Supplementary Materials

The following supporting information can be downloaded at: https://www.mdpi.com/article/10.3390/ijerph23080989/s1, File S1: Detailed Search Strategy; File S2: PRISMA Checklist [13]; File S3: Full references for curricula; Table S1: Overview of Programs (n = 42) and Curricula (n = 55) [22,41,42,43,48,49,50,51,87,88,89,90,91,92,93,94,95,96,97,98,99,100,101,102,103,104,105,106,107,108,109,110,111,112,113,114,115,116,117,118,119,120,121,122,123,124,125,126,127,128,129,130,131,132,133]; Table S2: Definitions of key topic areas in IPV prevention curricula; Table S3: Inclusion of key topic areas in curricula by program type; Table S4: Inclusion of key topic areas in curricula by population reached; Table S5: Curricula adaptation and common source materials (n = 55).

## Abbreviations

The following abbreviations are used in this manuscript:

IPV	Intimate partner violence
SRH	Sexual and reproductive health
ToC	Theory of change
VAC	Violence against children
VAW	Violence against women
VAWG	Violence against women and girls
