# Supplementary material for: A Scoping Review of IPV Prevention Curricula and Their Implementation in Low- and Middle-Income Countries: Implications for the Next Generation of Programs
Source: Int J Environ Res Public Health. 2026 Jul 29;23(8):989. doi: 10.3390/ijerph23080989 (PMC13512808; doi:10.3390/ijerph23080989)
Supplement: Supplementary file 1 [file ijerph-23-00989-s001.zip › Supplemental File 1 Detailed Search Strategy.pdf]

Supplementary File S1: Detailed Search Strategy

BACKGROUND

The focus of this scoping review was on the curricula used in intimate partner violence (IPV) prevention programs, rather than on studies of program effectiveness. As curricula are not systematically published or catalogued in any database, our aim was to identify and access as many IPV prevention curricula as possible to understand the current landscape. We designed a pragmatic search strategy, combining multiple complementary approaches developed through an iterative process, described in detail below. We share this level of detail in the hope that our process may be useful to other researchers facing similar challenges in identifying interventions or literature that are not systematically indexed or published.

This approach builds on an earlier phase of the project, which aimed to link curriculum content and implementation with evidence of program impact. Like the current review, that earlier phase combined a literature search with grey literature searching and consultation with experts, using approaches similar to those described in Strategy 2 of the current review, below. Only the earlier phase's literature search is described in detail here, as its findings most directly informed the design of the search strategy used for the current review.

Literature search conducted during the earlier project phase (2020–2021)

This search was conducted in 2020 in PubMed, Google, Google Scholar, and Scopus, to identify curriculum-based IPV prevention programs. The full PubMed search strategy is presented in Table 1.

Table 1. PubMed search strategy (earlier project phase, 2020)

Concept	Search Terms / Syntax
Concept #1 (IPV)	Intimate Partner Violence[mesh] OR Gender-Based Violence OR Intimate Partner Abuse*[tw] OR Intimate Partner violence[tw] OR Gender-Based Violence[tw]
Concept #2 (Curriculum)	Curriculum[mesh] OR workshop*[tw] OR training program*[tw] OR curricul*[tw] OR training[tw]
Concept #3 (LMIC filter)	PubMed LMIC filter (per World Bank, 2013): "developing country"[tiab] OR "developing countries"[tiab] OR "developing nation"[tiab] OR "developing nations"[tiab] OR "developing population"[tiab] OR "developing populations"[tiab] OR "developing world"[tiab] OR "less developed country"[tiab] OR "less developed countries"[tiab] OR "lesser developed country"[tiab] OR "under developed country"[tiab] OR "underdeveloped country"[tiab] OR "middle income country"[tiab] OR "middle income countries"[tiab] OR "low income country"[tiab] OR "low income countries"[tiab] OR "lower income country"[tiab] OR "underserved country"[tiab] OR "underserved population"[tiab] OR lmic[tiab] OR lmics[tiab] OR "third world"[tiab] OR Africa[tiab] OR Asia[tiab] OR Caribbean[tiab] OR "South America"[tiab] OR "Latin America"[tiab] OR "Central America"[tiab] OR "Eastern Europe"[tiab] OR [INSERT FULL LIST OF INDIVIDUAL LMIC COUNTRY NAMES, e.g., Afghanistan, Bangladesh, Brazil ... per the World Bank (2013) filter] OR Developing Countries[Mesh] OR Africa[Mesh:noexp] OR "Africa, Northern"[Mesh:noexp] OR "Africa South of the

	Sahara"[Mesh:noexp] OR Asia[Mesh:noexp] OR [INSERT FULL LIST OF INDIVIDUAL LMIC COUNTRY MeSH TERMS per the World Bank (2013) filter]

This earlier search indicated that literature searching alone was insufficient for identifying IPV prevention programs with curriculum-based components. This is largely because certain programs are not represented in the published literature, particularly those implemented by smaller national or local NGOs. This finding directly informed the design of the search strategy used for the current review, described below. Seventeen of the 55 curricula included in the current review were also identified during this earlier phase.

SEARCH STRATEGY FOR THE CURRENT REVIEW

Several years elapsed between the earlier phase of the project (2020–2021) and the current review, which began in late 2023 with a defined set of research questions focused on curriculum design, content, and implementation. The search for the current review was conducted between January 2024 and April 2025, to allow newer curricula to be included as they became available. Searches and screening for the current review were conducted by the first author. All authors contributed to expert consultations (Strategy 2) and to identifying relevant grey literature.

PROGRAM IDENTIFICATION AND SCREENING

Strategy 1: Literature Search of Existing Evidence Base

In the years between the earlier project phase and the current review, a wealth of new programs, evaluations, and evidence reviews had emerged. We used this expanded evidence base as the starting point for our current literature search strategy. We identified eight seed papers — published systematic or evidence reviews highly relevant to the focus of our review, published since 2020 (the start date of the earlier project phase). Several reviews were already known to us given our position in the field, and others were identified through web searches of Google Scholar on January 19, 2024, using combinations of the terms ‘violence against women’, ‘intimate partner violence’, and ‘systematic review’, with publication dates limited to 2020 or later. We were also given access to one review prior to its publication. These reviews are references 7, 9, 10, 14–18 in the full manuscript.

All programs cited in these reviews were documented in an Excel tracking file. After removing duplicates, the reviews cited 182 unique programs, of which 35 were assessed as eligible after screening (described below).

Strategy 2: Search of Grey Literature, Websites, and Consultation with Experts

As professionals working in this field, we were already aware of a number of eligible programs and related literature through our own knowledge, networks, and the earlier project phase, without the need for formal searching. In addition to this existing knowledge, we actively identified further programs through: a) web searches to identify grey literature, b) hand-searching known websites of violence prevention funders and implementers, and c) consultation with known experts. Each of these methods is described below.

All programs identified were documented in the same Excel tracking file used for Strategy 1, and duplicates with programs already identified under Strategy 1 were noted. In total, this strategy identified 95 additional programs, of which 17 were assessed as eligible after screening.

Grey literature search

In addition to the programs and literature already known to us through our professional networks (noted above), multiple combinations of relevant search terms were used to search the Google search engine. Search terms included: 'violence against women', 'intimate partner violence', 'gender-based violence', 'violence prevention', 'curriculum', 'manual', and 'group-based'. Given the volume of results returned by general web searches, it was not feasible to review the full return for each search; relevance judgments were made by the reviewers based on an initial subset of results.

Websites of known funders and program developers

We hand-searched websites of known violence prevention funders and program developers, building on an initial search conducted during the earlier project phase in 2020. This search was expanded during the current review. Websites included:

- EngenderHealth
- Equimundo (formerly Promundo-US)
- International Rescue Committee
- MenEngage Alliance
- Sonke Gender Justice
- Partners for Prevention (site no longer active)
- Prevention Collaborative
- Population Council
- Sexual Violence Research Initiative
- UN Women
- UNFPA
- UNDP
- UNICEF
- What Works to Prevent Violence Against Women and Girls

Consultation with experts

We used our position within the field to contact experts to identify additional programs and curricula. Individuals likely to be aware of relevant programs, including unpublished ones, were considered content experts; these included known violence prevention funders, researchers, practitioners, and program developers or implementers. Contacts were made throughout the search period, as we became aware of new programs or made new contacts.

Experts sometimes shared program documents or curricula directly, or put us in touch with those who could; otherwise, we searched for relevant documents using Google Search, using the program or organization name and country of implementation as search terms.

Strategy 3: Reports from the United Nations Trust Fund to End Violence Against Women

The third search strategy was designed to identify more programs implemented by smaller non-governmental organizations, which are less represented in the published literature. Strategies 1 and 2 tended to identify programs developed or implemented by (or with the involvement of) international NGOs and institutions.

We decided to explore programs funded by the United Nations Trust Fund to End Violence against Women and Girls (UN Trust Fund), which provides grants to organizations working to prevent and respond to violence against women and girls. We downloaded and reviewed 153 final evaluation reports from UNTF grantees. These were accessed from the UNTF website on December 4, 2024.[1]

All programs cited in the UNTF reports were documented in the same Excel tracking file used for Strategies 1 and 2, and duplicates with programs already identified were noted. This strategy identified 16 additional eligible programs after screening.

Screening Process Applied to All Three Search Strategies

All programs identified through the three search strategies were documented in an Excel file, updated as the search evolved. Programs were screened against our eligibility criteria in the following order, and adherence to each criterion was documented:

- Was the program implemented in a low- or middle-income country (LMIC), as defined by the World Bank?
- Was IPV prevention a primary aim, as opposed to prevention of other forms of violence against women and girls, or response to violence?
- Did the program include a group-based component guided by a structured curriculum?
- Was the program implemented after 2000?[2]

For the evidence reviews (Strategy 1), screening was based on information reported in the reviews (which primarily allowed us to assess LMIC eligibility), and, most often, by accessing the original studies or papers cited in the reviews through backward citation searching.

For the UN Trust Fund final evaluation reports (Strategy 3), screening based on the reports alone was not always possible. Where feasible, UN Trust Fund colleagues put us in touch with the original program implementers (in January 2025) whom we contacted by email to assess eligibility and, where relevant, arrange interviews. Excel was used to document contact attempts, responses received, programs/curricula identified, and reminder messages sent to non-respondents. Contacting some program implementers was difficult due to staff turnover at the UNTF or among grantee organizations (e.g., non-functioning email addresses, or new staff unfamiliar with the program).

[1] https://untf.unwomen.org/en/digital-library/publications
[2] Our search strategies identified very few programs implemented prior to 2000; these were mostly identified through contacts or grey literature.

Table 2. Programs assessed and identified as eligible, by search strategy

Strategy	Source(s)	Programs Assessed	Eligible Programs Identified
Strategy 1	8 systematic/evidence reviews published since 2020 (182 unique programs cited, after removing duplicates)	182	35
Strategy 2	Grey literature, professional contacts, and violence prevention funder/implementer websites	95	17
Strategy 3	UN Trust Fund to End Violence against Women and Girls evaluation reports	153	16
Total		430	68

OBTAINING CURRICULA FROM ELIGIBLE PROGRAMS

Following the identification of eligible programs, we sought to obtain the IPV prevention curricula used in these programs. This process was ongoing as our search strategy evolved and newer programs were identified. We sought to obtain the curricula, as well as relevant documentation of program implementation, using the following strategies:

- Google Search using relevant search terms (i.e., program or curriculum name, country of implementation, program implementer name).
- Web search of the program developer/implementer's website.
- Contacting program implementers, evaluators, or funders by email to request a copy of the curriculum or to be put in touch with someone who could provide information or access.

We documented in Excel whether we were able to (where relevant) make contact with program developers, whether the curriculum was available in English, French, or Spanish, and whether we were able to obtain the curriculum.

Of the 68 eligible programs, curricula were obtained from 45 programs; some programs included multiple curricula designed for different populations. Curricula were then screened to determine whether they met our eligibility criteria, namely adherence to our ‘curriculum’ definition. Three programs were excluded because they did not meet this definition, and these determinations were documented.

The final review includes 55 curricula from 42 programs.
