# Supplementary material for: A Scoping Review of IPV Prevention Curricula and Their Implementation in Low- and Middle-Income Countries: Implications for the Next Generation of Programs
Source: Int J Environ Res Public Health. 2026 Jul 29;23(8):989. doi: 10.3390/ijerph23080989 (PMC13512808; doi:10.3390/ijerph23080989)
Supplement: Supplementary file 1 [file ijerph-23-00989-s001.zip › Supplemental File 3 Full references for curricula.pdf]

Supplemental File S3: Full references for curricula

Note: Several curricula and their supporting documents were shared with the study team but are not publicly available. Where resources are publicly available, we have included a hyperlink and the last date the link was accessed. Some resources were previously but are no longer available online unfortunately.

Bandebereho

1. Rwanda Men's Resource Center (RWAMREC); Promundo-US. *Facilitator's Manual: Engaging fathers in gender equality, maternal and child health, caregiving and violence prevention*; RWAMREC: Kigali, Rwanda, 2014. Available online: https://prevention-collaborative.org/wp-content/uploads/2021/08/Bandebereho-Facilitators-Manual-Fathers.pdf (accessed on 23 April 2026)
2. Rwanda Men's Resource Center (RWAMREC); Promundo-US. *Facilitator's Manual: Engaging young women in sexual and reproductive health and gender equality*; RWAMREC: Kigali, Rwanda, 2014. Available online: https://www.mencare.org/wp-content/uploads/2015/08/Bandebereho-Facilitators-Manual-Young-Women.pdf (accessed on 23 April 2026)
3. Rwanda Men's Resource Center (RWAMREC); Promundo-US. *Facilitator's Manual: Engaging young men in sexual and reproductive health and gender equality*; RWAMREC: Kigali, Rwanda, 2014. Available online: https://www.mencare.org/wp-content/uploads/2015/08/Bandebereho-Facilitators-Manual-Young-Men.pdf (accessed on 23 April 2026)
4. Doyle, K.; Levtov, R.G.; Barker, G.; Bastian, G.G.; Bingenheimer, J.B.; Kazimbaya, S.; Nzabonimpa, A.; Pulerwitz, J.; Sayinzoga, F.; Sharma, V.; et al. Gender-Transformative Bandebereho Couples' Intervention to Promote Male Engagement in Reproductive and Maternal Health and Violence Prevention in Rwanda: Findings from a Randomized Controlled Trial. PLoS ONE 2018, 13, e0192756, https://doi.org/10.1371/journal.pone.0192756.
5. Doyle, K.; Levtov, R.G.; Karamage, E.; Rakshit, D.; Kazimbaya, S.; Sayinzoga, F.; Sibomana, H.; Ngayaboshya, S.; Rutayisire, F.; Barker, G. Long-Term Impacts of the Bandebereho Programme on Violence against Women and Children, Maternal Health-Seeking, and Couple Relations in Rwanda: A Six-Year Follow-up of a Randomised Controlled Trial. eClinicalMedicine 2023, 64, 102233, https://doi.org/10.1016/j.eclinm.2023.102233.

Becoming One

1. International Rescue Committee (IRC); World Vision. *Becoming One: A guide for faith leaders*; International Rescue Committee: New York, NY, USA n.d. Available online: https://prevention-collaborative.org/wp-content/uploads/2021/08/FL-Guide-with-Violence-Content-English.pdf (accessed on 22 April 2026).
2. International Rescue Committee (IRC); World Vision. *Becoming One: Couples' Guide*; International Rescue Committee: New York, NY, USA n.d.
3. Prevention Collaborative. *Programme Summary: Becoming One, A Religious Couples' Counselling Programme to Prevent Violence in Western Uganda*; Prevention Collaborative: 2020. Available online: https://prevention-collaborative.org/wp-content/uploads/2021/08/Programme-Summary_Becoming-One-1.pdf (accessed on 22 April 2026)
4. Boyer, C.; Levy Paluck, E.; Annan, J.; Nevatia, T.; Cooper, J.; Namubiru, J.; Heise, L.; Lehrer, R. Religious Leaders Can Motivate Men to Cede Power and Reduce Intimate Partner Violence: Experimental Evidence from Uganda. Proc. Natl. Acad. Sci. U.S.A. 2022, 119, e2200262119, https://doi.org/10.1073/pnas.2200262119.

Change Starts at Home

1. Equal Access International; South African Medical Research Council (SAMRC). *BIG Change Curriculum: A Discussion Guide for Prevention of Intimate Partner Violence*; Equal Access International; Kathmandu, Nepal, 2017. Available online: https://www.whatworks.co.za/resources/item/494-big-change-curriculum (accessed on 22 April 2026)
2. McGhee, S.; Shrestha, B.; Ferguson, G.; Shrestha, P.N.; Bergenfeld, I.; Clark, C.J. "Change Really Does Need to Start From Home": Impact of an Intimate Partner Violence Prevention Strategy Among Married

Couples in Nepal. J Interpers Violence 2021, 36, NP7840–NP7867, https://doi.org/10.1177/0886260519839422.
3. Clark, C.J.; Shrestha, B.; Ferguson, G.; Shrestha, P.N.; Calvert, C.; Gupta, J.; Batayeh, B.; Bergenfeld, I.; Oakes, J.M. Impact of the Change Starts at Home Trial on Women's Experience of Intimate Partner Violence in Nepal. SSM - Population Health 2020, 10, 100530, https://doi.org/10.1016/j.ssmph.2019.100530.
4. Clark, C.J.; Bergenfeld, I.; Batayeh, B.; Ferguson, G.; Shrestha, B.; Shrestha, P.N. *Intimate Partner Violence in Nepal: Change Starts at Home Study. Evidence Brief*; What Works to Prevent Violence against Women and Girls: October 2019. Available online: https://www.whatworks.co.za/resources/item/676-intimate-partner-violence-in-nepal-change-starts-at-home-study (accessed on 22 April 2026)
5. Prevention Collaborative. *Programme Summary: Change Starts at Home, Nepal*; Prevention Collaborative: 2024. Available online: https://prevention-collaborative.org/wp-content/uploads/2024/12/Ps-Change-Starts-At-Home-FINAL-DEC-2024.pdf (accessed on 22 April 2026)

Community-based Prevention of Violence against Refugee Women and Girls

1. HIAS Refugee Trust of Kenya. *Discussion Guide*; HIAS Kenya: Nairobi, Kenya, n.d.
2. HIAS Refugee Trust of Kenya. *Facilitator's Guide*; HIAS Kenya: Nairobi, Kenya, n.d.
3. UN Women; HIAS Kenya; The Leviathan. *End of Project Evaluation Report. Community-based Prevention of Violence against Refugee Women and Girls in Nairobi Project*; UN Trust Fund to End Violence Against Women and Girls: 25 October 2022. Available online: https://untf.unwomen.org/sites/default/files/2023-07/17._hias_kenya_endline_evaluation_report_untf_final_clean_copy_compressed.pdf (accessed on 23 April 2026)

Do Kadam Barabari Ki Ore

4. CEDPA India; Population Council. *Do Kadam: Barabari Ki Ore — Peer Mentors Module for SHG Women and Husband Peer Mentors*; CEDPA India: New Delhi, India, n.d.
5. CEDPA India. *Peer Mentors Module for NYKS Clubs*; CEDPA India: New Delhi, India, n.d.
6. Population Council; Centre for Catalyzing Change; London School of Hygiene & Tropical Medicine. *Enabling Adolescent Boys to Adopt Attitudes that Espouse Gender Equality and Oppose Violence Against Women and Girls: Evidence from Rural Bihar*; Population Council: New Delhi, India, 2017.
7. Population Council; Centre for Catalyzing Change; London School of Hygiene & Tropical Medicine. *Reducing Violence Against Women and Girls in India: Lessons from the Do Kadam Programme*; Population Council: New Delhi, India, 2017.
8. Population Council; Centre for Catalyzing Change; London School of Hygiene & Tropical Medicine. *The Effect of a Gender Transformative Life Skills Education and Sports-Coaching Programme on the Attitudes and Practices of Adolescent Boys and Young Men in Bihar*; Population Council: New Delhi, India, 2017.
9. Population Council; Centre for Catalyzing Change; London School of Hygiene & Tropical Medicine. *Empowering Women and Addressing Marital Violence Through Self-Help Groups: Evidence from Rural Bihar*; Population Council: New Delhi, India, 2017.
10. Santhya, K.G.; Francis Zavier, A.J. Long-Term Impact of Exposure to a Gender-Transformative Program Among Young Men: Findings From a Longitudinal Study in Bihar, India. Journal of Adolescent Health 2022, 70, 634–642, https://doi.org/10.1016/j.jadohealth.2021.10.041.
11. Jejeebhoy, S.J.; Santhya, K. Preventing Violence against Women and Girls in Bihar: Challenges for Implementation and Evaluation. Reproductive Health Matters 2018, 26, 92–108, https://doi.org/10.1080/09688080.2018.1470430.
12. Jejeebhoy, S.; Acharya, R.; Pandey, N.; Santhya, K.G.; Zavier, A.J.; Singh, S.; Saxena, K.; Gogoi, A.; Joshi, M.; Ojha, S. *The Effect of a Gender Transformative Life Skills Education and Sports-Coaching Programme on the Attitudes and Practices of Adolescent Boys and Young Men in Bihar*; Population Council: New Delhi, India, 2017.
13. Gupta, A.K.; Santhya, K.G. Promoting Gender Egalitarian Norms and Practices Among Boys in Rural India: The Relative Effect of Intervening in Early and Late Adolescence. Journal of Adolescent Health 2020, 66, 157–165, https://doi.org/10.1016/j.jadohealth.2019.03.007.

Economic and Social Empowerment (EA$E)

1. International Rescue Committee. *EA$E Discussion Series: Facilitator Guide*; International Rescue Committee: New York, NY, USA, 2012.
2. International Rescue Committee. *Economic and Social Empowerment (EA$E): Program Implementation Manual*; International Rescue Committee: New York, NY, USA, 2012.
3. International Rescue Committee. *Economic and Social Empowerment to Reduce Violence against Women: Research Brief*; International Rescue Committee: New York, NY, USA, 2014.
4. Gupta, J.; Falb, K.L.; Lehmann, H.; Kpebo, D.; Xuan, Z.; Hossain, M.; Zimmerman, C.; Watts, C.; Annan, J. Gender Norms and Economic Empowerment Intervention to Reduce Intimate Partner Violence against Women in Rural Côte d'Ivoire: A Randomized Controlled Pilot Study. BMC Int Health Hum Rights 2013, 13, 46, https://doi.org/10.1186/1472-698X-13-46.
5. Falb, K.L.; Annan, J.; Kpebo, D.; Cole, H.; Willie, T.; Xuan, Z.; Raj, A.; Gupta, J. Differential Impacts of an Intimate Partner Violence Prevention Program Based on Child Marriage Status in Rural Côte d'Ivoire. Journal of Adolescent Health 2015, 57, 553–558, https://doi.org/10.1016/j.jadohealth.2015.08.001.
6. Falb, K.L.; Annan, J.; King, E.; Hopkins, J.; Kpebo, D.; Gupta, J. Gender Norms, Poverty and Armed Conflict in Cote D'Ivoire: Engaging Men in Women's Social and Economic Empowerment Programming. Health Education Research 2014, 29, 1015–1027, https://doi.org/10.1093/her/cyu058.

Engaging Men in Accountable Practice

1. International Rescue Committee. *Preventing Violence Against Women and Girls: Engaging Men Through Accountable Practice — Part 1: Introductory Guide*; International Rescue Committee: New York, NY, USA, 2013.
2. International Rescue Committee. *Preventing Violence Against Women and Girls: Engaging Men Through Accountable Practice — Part 2: Training Guide*; International Rescue Committee: New York, NY, USA, 2014.
3. International Rescue Committee. *Preventing Violence Against Women and Girls: Engaging Men Through Accountable Practice — Part 3: Implementation Guide*; International Rescue Committee: New York, NY, USA, 2014.
4. Vaillant, J.; Koussoubé, E.; Roth, D.; Pierotti, R.; Hossain, M.; Falb, K.L. Engaging Men to Transform Inequitable Gender Attitudes and Prevent Intimate Partner Violence: A Cluster Randomised Controlled Trial in North and South Kivu, Democratic Republic of Congo. BMJ Glob Health 2020, 5, e002223, https://doi.org/10.1136/bmjgh-2019-002223.
5. Gurbuz Cuneo, A.; Vaillant, J.; Koussoubé, E.; Pierotti, R.S.; Falb, K.; Kabeya, R. Prevention, Cessation, or Harm Reduction: Heterogeneous Effects of an Intimate Partner Violence Prevention Program in Eastern Democratic Republic of the Congo. PLoS ONE 2023, 18, e0282339, https://doi.org/10.1371/journal.pone.0282339.

Engaging with Faith Groups to Prevent Violence Against Women and Girls in conflict-Affected Communities

1. Deepan, P. *Community Dialogues: Promoting respectful relationships and equitable communities*; Tearfund: Middlesex, United Kingdom, 2016.
2. Deepan, P. *Transforming Masculinities: A training manual for Gender Champions*; Tearfund: Middlesex, United Kingdom, 2017.
3. Thomson, S.; Quirke, F.; Deepan, P.; Martin, L.; Nkusi, S.; Baghuma, U.L.; Bezzolato, E.; McLarnon-Silk, C. *Transforming Masculinities Implementation Guide: A step by step guide for implementing Transforming Masculinities projects*; Tearfund: Middlesex, United Kingdom, 2021.
4. Le Roux, E.; Bowers-Du Toit, N. Men and Women in Partnership: Mobilizing Faith Communities to Address Gender-Based Violence. Diaconia 2017, 8, 23–37, https://doi.org/10.13109/diac.2017.8.1.23.
5. Le Roux, E.; Corboz, J.; Scott, N.; Sandilands, M.; Lele, U.B.; Bezzolato, E.; Jewkes, R. Engaging with Faith Groups to Prevent VAWG in Conflict-Affected Communities: Results from Two Community Surveys in the DRC. BMC Int Health Hum Rights 2020, 20, 27, https://doi.org/10.1186/s12914-020-00246-8.
6. Palm, S.; Le Roux, E.; Bezzolato, E.; Deepan, P.; Corboz, J.; Lele, U.; O'Sullivan, V.; Jewkes, R. *Rethinking Relationships: Moving from Violence to Equality. What Works to Prevent Violence against Women and Girls in the Democratic Republic of Congo*; Tearfund: Middlesex, United Kingdom, 2019.

Gender Responsive School Model Pilot Project

1. Plan International Vietnam. *Training Manual on School Related Gender Based Violence Prevention and Response for Teachers of Lower Secondary Schools*; Plan International Vietnam: Hanoi, Vietnam, n.d.
2. Plan International Vietnam. *Training Manual on School Related Gender Based Violence Prevention and Response for Teachers of High Schools*; Plan International Vietnam: Hanoi, Vietnam, n.d.
3. Plan International Vietnam. *Guidelines for Developing a Safe, Friendly and Equal Environment in Schools: Reference Document*; Plan International Vietnam: Hanoi, Vietnam, n.d.
4. Plan International Vietnam. *Gender Responsive School Briefing Note*; Plan International Vietnam: Hanoi, Vietnam, n.d.
5. Singh, N.; Ta, T.H.; Phung, T.Y. *Final Evaluation Report: The Gender Responsive School Pilot Model Project*; Plan International Vietnam: Hanoi, Vietnam, 2017.
6. Programme staff member, Plan International Vietnam, Vietnam. Remote interview, 2025.

Get Into Rugby Plus

1. Oceania Rugby, Child Fund, UN Women. *Get Into Rugby Plus Sessions 1-16*; Oceania Rugby: Surry Hills, NSW, Australia, n.d.
2. Oceania Rugby and UN Women. Rugby Coaches to Promote Gender Equality and Prevention of Violence Against Women and Girls through Get Into Rugby PLUS. 30 April 2019. Available online: https://asiapacific.unwomen.org/en/news-and-events/stories/2019/04/rugby-coaches-to-promote-gender-equality-and-prevention-of-violence-against-women-and-girls (accessed on 22 April 2026)
3. King, A. In Fiji, Teaching Life Skills and Changing Mindsets Through Rugby. UN Women. 26 February 2020. Available online: https://www.unwomen.org/en/news/stories/2019/6/feature-in-fiji-changing-mindsets-through-rugby (accessed on 22 April 2026).
4. Uluinakauvadra, T. Get Into Rugby Plus: Transforming Lives Through Sport. Presented at the Asia-Pacific Forum on Sustainable Development (APFSD), Bangkok, Thailand, 25 February 2025.

Ghya Bharari Ekatra

1. Sahay, S.; Kalokhe, A. *Fly High Together*; ICMR National AIDS Research Institute and Emory University: Pune, India, 2018.
2. Kalokhe, A.S.; Iyer, S.; Katendra, T.; Gadhe, K.; Kolhe, A.R.; Paranjape, A.; Del Rio, C.; Stephenson, R.; Sahay, S. Primary Prevention of Intimate Partner Violence Among Recently Married Dyads Residing in the Slums of Pune, India: Development and Rationale for a Dyadic Intervention. JMIR Res Protoc 2019, 8, e11533, https://doi.org/10.2196/11533.
3. Kalokhe, A.S.; Iyer, S.; Gadhe, K.; Katendra, T.; Kolhe, A.; Rahane, G.; Stephenson, R.; Sahay, S. A Couples-Based Intervention (Ghya Bharari Ekatra) for the Primary Prevention of Intimate Partner Violence in India: Pilot Feasibility and Acceptability Study. JMIR Form Res 2021, 5, e26130, https://doi.org/10.2196/26130.

HERrespect

1. Yu, M.; Wan, J.; Jewkes, R.; Willian, S.; Huq, N.; Rahman, T. *HERrespect Curriculum: Management*; Business for Social Responsibility: San Francisco, CA, USA; South African Medical Research Council: Pretoria, South Africa, 2018.
2. Yu, M.; Wan, J.; Jewkes, R.; Willian, S.; Huq, N.; Rahman, T. *HERrespect Curriculum: Male Workers*; Business for Social Responsibility: San Francisco, CA, USA; South African Medical Research Council: Pretoria, South Africa, 2018.
3. Yu, M.; Wan, J.; Jewkes, R.; Willian, S.; Huq, N.; Rahman, T. *HERrespect Curriculum: Women Workers*; Business for Social Responsibility: San Francisco, CA, USA; South African Medical Research Council: Pretoria, South Africa, 2018.
4. Business for Social Responsibility and South African Medical Research Council. *About the HERrespect Curriculum*; BSR and SAMRC: 2018.

5. Naved, R.T.; Mamun, M.A.; Parvin, K.; Willan, S.; Gibbs, A.; Jewkes, R. Learnings from the Evaluation of HERrespect: A Factory-Based Intervention to Prevent Intimate Partner and Workplace Violence against Female Garment Workers in Bangladesh. Global Health Action 2021, 14, 1868960, https://doi.org/10.1080/16549716.2020.1868960.

Humqadam

1. Rozan. *Engaging with Boys and Young Men to Address Gender Based Violence and Masculinities. A training module*; Rozan: Islamabad, Pakistan, 2011.
2. Marcus, R.; Stavropoulou, M.; Archer-Gupta, N. *Programming with Adolescent Boys to Promote Gender-Equitable Masculinities. A rigorous review*; Gender and Adolescence: Global Evidence (GAGE): December 2018.
3. Rozan. *Report. Evaluation Study: Working with Men and Boys on Prevention of GBV*; Rozan: Islamabad, Pakistan, January 2012.

Intervention with Microenterprise for Aids and Gender Equity (IMAGE) Study

1. Rural AIDS & Development Action Research Program (RADAR), School of Public Health University of the Witwatersrand, Small Enterprise Foundation (SEF). *Sisters for Life: Gender & HIV Training Manual*; RADAR, University of Witswatersrand: Johannesburg, South Africa; SEF: Tzaneen, South Africa, 2006.
2. Hatcher, A.; De Wet, J.; Bonell, C.P.; Strange, V.; Phetla, G.; Proynk, P.M.; Kim, J.C.; Morison, L.; Porter, J.D.H.; Busza, J.; et al. Promoting Critical Consciousness and Social Mobilization in HIV/AIDS Programmes: Lessons and Curricular Tools from a South African Intervention. Health Education Research 2011, 26, 542–555, https://doi.org/10.1093/her/cyq057.
3. Kim, J.C.; Watts, C.H.; Hargreaves, J.R.; Ndhlovu, L.X.; Phetla, G.; Morison, L.A.; Busza, J.; Porter, J.D.H.; Pronyk, P. Understanding the Impact of a Microfinance-Based Intervention on Women's Empowerment and the Reduction of Intimate Partner Violence in South Africa. Am J Public Health 2007, 97, 1794–1802, https://doi.org/10.2105/AJPH.2006.095521.
4. Kim, J.; Ferrari, G.; Watts, C.; Hargreaves, J.; Morison, L.; Phetla, G.; Porter, J.; Pronykh, P.; Abramsky, T. Assessing the Incremental Effects of Combining Economic and Health Interventions: The IMAGE Study in South Africa. Bull World Health Org 2009, 87, 824–832, https://doi.org/10.2471/BLT.08.056580.
5. Pronyk, P.M.; Hargreaves, J.R.; Kim, J.C.; Morison, L.A.; Phetla, G.; Watts, C.; Busza, J.; Porter, J.D. Effect of a Structural Intervention for the Prevention of Intimate-Partner Violence and HIV in Rural South Africa: A Cluster Randomised Trial. The Lancet 2006, 368, 1973–1983, https://doi.org/10.1016/S0140-6736(06)69744-4.
6. Kim, J.; Hargreaves, J.; Makhubele, B.M.; Mashaba, K.; Morison, L.; Motsei, M.; Peters, C.; Porter, J.; Pronyk, P.; Watts, C. *Social Interventions for HIV/AIDS Intervention with Microfinance for AIDS and Gender Equity. IMAGE Study Monograph 2: Intervention*; Rural AIDS and Development Action Research Program: November 2002.

Improved Action Against Sexual and Gender-based Violence and Harmful Practices

1. Scripture Union West Africa (SUWA). *Community Dialogues Facilitators' Manual on Sexual and Gender Based Violence. A Manual of SUWA*; SUWA: Ibadan, Oyo State, Nigeria, n.d.
2. Mershak, P.; Chinonso, K. *Final Report: SUWA End-Term Evaluation;* Scripture Union West Africa (SUWA), UN Women, UN Trust Fund to End Violence Against Women: March 2023.
3. Program staff members, Scripture Union West Africa, Nigeria. Virtual interview, 2025.

Indashyikirwa

1. Yaker, R. *Couple Curriculum Training Module*; CARE Rwanda and Rwanda Men's Resource Center (RWAMREC): Kigali, Rwanda, 2018.

2. Dunkle, K.; Stern, E.; Chatterji, S.; Heise, L. Effective Prevention of Intimate Partner Violence through Couples Training: A Randomised Controlled Trial of Indashyikirwa in Rwanda. BMJ Glob Health 2020, 5, e002439, https://doi.org/10.1136/bmjgh-2020-002439.
3. Stern, E.; Nyiratunga, R. A Process Review of the Indashyikirwa Couples Curriculum to Prevent Intimate Partner Violence and Support Healthy, Equitable Relationships in Rwanda. Social Sciences 2017, 6, 63, https://doi.org/10.3390/socsci6020063.
4. What Works to Prevent Violence against Women and Girls. *Impact of Indashyikirwa. An innovative programme to reduce partner violence in rural Rwanda – Evidence Brief;* What Works to Prevent Violence against Women and Girls: August 2019.
5. Stern, E. *Programme Summary: The Indashyikirwa Programme, Rwanda*; Prevention Collaborative: January 2019.

Managing Work and Life

1. Rolleri, L.; Kotsadam, A.; Villanger, E. *Managing Work and Life*: *A Gender Transformative Intimate Partner Violence Prevention Course for Women in Ethiopia*; Chr. Michelsen Institute: Bergen, Norway, 2017.
2. Kotsadam, A.; Villanger, E. Effects of Empowerment Training on Attitudes, Agency and Intimate Partner Violence in Ethiopia. Unpublished manuscript.

Masculinité, Famille, et Foi

1. Deepan, P.; McLarnon-Silk, C.; Quirke, F. *Community Dialogues: Promoting respectful relationships and equitable communities with sessions on child spacing*; Tearfund, Middlesex, United Kingdom; Institute of Reproductive Health, Georgetown, Washington, D.C., United States: 2020. Available online: https://learn.tearfund.org/en/resources/tools-and-guides/community-dialogues-with-sessions-on-child-spacing (accessed on 23 April 2026)
2. Thomson, S.; Deepan, P.; Martin, L.; Quirke, F.; Nkusi, S.; Baghuma, U.L.; Bezzolato, E.; McLarnon-Silk, C.; *Transformer les Masculinités: Guide de mise en oeuvre. Guide étape par étape pour la mise en oeuvre des projets Transformer les masculinités*; Tearfund, Middlesex, United Kingdom; Institute of Reproductive Health, Georgetown, Washington, D.C., United States: 2022. Available online: https://learn.tearfund.org/fr-fr/resources/series/changing-gender-norms-transforming-masculinities/transforming-masculinities-implementation-guide (accessed on 23 April 2026)
3. Institute for Reproductive Health (IRH) and Center for Child and Human Development, Georgetown University. *Transforming Masculinities/Masculinité, Famille, et Foi Intervention – Endline Quantitative Research Report;* Institute for Reproductive Health (IRH) and Center for Child and Human Development, Georgetown University with the United States Agency for International Development (USAID): Washington, D.C., September 2020. Available online: https://www.irh.org/wp-content/uploads/2020/09/Passages_MFF-Quantitative-End-Line-Report_Final_09.30.2020.pdf (accessed on 23 April 2026)
4. Institute for Reproductive Health (IRH) and Center for Child and Human Development, Georgetown University. *Masculinite, Famille, et Foi: End of Project Report;* Institute for Reproductive Health (IRH) and Center for Child and Human Development, Georgetown University for the United States Agency for International Development (USAID): Washington, D.C., April 2021. Available online: https://www.irh.org/wp-content/uploads/2021/04/MFF-EOP-REPORT_FINAL_MARCH_2021-5.3.21.pdf (accessed on 23 April 2026)

Men in Partnership

1. International Rescue Committee. *Men and Women in Partnership*; International Rescue Committee Ivory Coast: 2010.
2. Hossain, M.; Zimmerman, C.; Kiss, L.; Abramsky, T.; Kone, D.; Bakayoko-Topolska, M.; Annan, J.; Lehmann, H.; Watts, C. Working with Men to Prevent Intimate Partner Violence in a Conflict-Affected Setting: A Pilot Cluster Randomized Controlled Trial in Rural Côte d'Ivoire. BMC Public Health 2014, 14, 339, https://doi.org/10.1186/1471-2458-14-339.

I Can Say No

1. Andras, R.; Ileš, M.; Pavlović, S.; Mészáros, G.; Dés, F.; Cesar, S.; Bijelić, N. *Capacity Training Manual to raise awareness and contribute to ending Cyber Sexual and Gender-Based Violence in Youth intimate partner relationships;* Fundación Privada INDERA: Barcelona, Spain, 2020.
2. Program staff member, Autonomni ženski centar, Serbia. Virtual interview, 2025.

Parenting for Respectability

1. Makerere University – Child Health and Development Centre (CHDC). *Parenting for Respectability Programme Facilitator's Manual;* Child Health and Development Centre: Kampala, Uganda, 2019.
2. Wight, D.; Sekiwunga, R.; Namutebi, C.; Zalwango, F.; Siu, G.E. A Ugandan Parenting Programme to Prevent Gender-Based Violence: Description and Formative Evaluation. Research on Social Work Practice 2022, 32, 448–464, https://doi.org/10.1177/10497315211056246.
3. Siu, G.; Nsubuga, R.N.; Lachman, J.M.; Namutebi, C.; Sekiwunga, R.; Zalwango, F.; Riddell, J.; Wight, D. The Impact of the Parenting for Respectability Programme on Violent Parenting and Intimate Partner Relationships in Uganda: A Pre-Post Study. PLoS ONE 2024, 19, e0299927, https://doi.org/10.1371/journal.pone.0299927.
4. Makerere University – Child Health and Development Centre (CHDC). *Ugandan Parenting for Respectability Implementation Science Evaluation;* Makerere University – Child Health and Development Centre (CHDC): Kampala, Uganda, n.d. Available online: https://prevention-collaborative.org/wp-content/uploads/2023/01/230111-GPI-UPRISE-Uganda-Policy-brief-V3.pdf (accessed on 23 April 2026)

Planim Save, Kamap Strongpela

1. Nazareth Centre for Rehabilitation (NCFR). *Planim Save, Kamap Strongpela Curriculum: A guide for promoting community social cohesion & security in Bougainville*; Nazareth Centre for Rehabilitation: Bougainville, Papua New Guinea, 2017.
2. Gevers, A.; Marin, C.; Taylor, K.; Kelly-Hanku, A.; Garasu, L.; Otim, J. *A Quantitative Evaluation of Planim Save Kamp Strongpela Intervention to Prevent Gender Based Violence and Build Peace in Bougainville, Papua New Guinea;* Partners for Prevention, UN Women, Nazareth Centre for Rehabilitation, PNG Institute of Medical Research: 2017. Available online: https://asiapacific.unwomen.org/en/digital-library/publications/2025/05/a-quantitative-evaluation-of-planim (accessed on 23 April 2026)
3. Gevers, A.; Taylor, K. *Promising Strides toward Ending Violence against Women and Girls in the Asia and the Pacific Region: Results from Partners for Prevention Pilot Interventions in Four Countries;* Partners for Prevention, UN Women, UNFPA, UN Volunteers, UNDP: February 2018. Available online: https://prevention-collaborative.org/wp-content/uploads/2024/02/Gevers-2018-Promising-strides-towards-ending-VAWG-in-the-Asia-.pdf (accessed on 23 April 2026)
4. Kelly-Hanku, A.; Mek, A.; Trumb, R.N. *Planim Save Kamap Strongpela: A qualitative endline study of an intervention to build peace and reduce gender based violence in South Bougainville;* PNG Institute of Medical Research, University of South Wales Sydney, Kirby Institute, Nazareth Centre for Rehabilitation, Partners for Prevention, UN Women: 2017. Available online: https://asiapacific.unwomen.org/en/digital-library/publications/2017/06/planim-save-kamap-strongpela (accessed on 23 April 2026)
5. Robertson, L.; Pagau, K. *End of Project Evaluation Report: Evaluation of Equality for Progress (E4P) and Planim Save Kamap Strongpela (Plant Knowledge, Grow Strong);* UN Women, UNICEF, UNDP, Partners for Prevention Regional Joint Programme: 16 April 2017. Available online: https://www.un.org/peacebuilding/sites/www.un.org.peacebuilding/files/documents/png_april_2017_-_evaluation_of_gender_promotion_initiative_2_projects.pdf (accessed on 23 April 2026)
6. Partners for Prevention. *Planim Save, Kamap Strongpela*. Partners for Prevention: 2017.
7. Moussa, J., Endut, N. *Evaluation of Partners for Prevention Regional Joint Programme for Violence against Women and Girls Prevention in Asia and the Pacific Phase II 2014 – 2017;* Partners for Prevention: April 2018. Available online: https://erc.undp.org/evaluation/documents/download/12031 (accessed on 23 April 2026)

Program Abb

1. Equimundo and ABAAD. *Program ABB-Early Childhood Development – A manual Promoting Positive Parenting and Strengthening Active Father's Involvement in Early Childhood Development;* Equimundo: Washington, D.C. USA.; ABAAD: Beirut, Lebanon, 2020. Available online:

https://www.equimundo.org/wp-content/uploads/2024/02/Program-ABB-EN-2024.pdf (accessed on 23 April 2026)
2. Promundo-US and ABAAD. *The Pilot of Program P-ECD in Lebanon: Evaluation Results and Lessons Learned;* Washington, DC: Promundo: 2019. Available online: https://www.equimundo.org/resources/the-pilot-of-program-p-ecd-in-lebanon-evaluation-results-and-lessons-learned/ (accessed on 23 April 2026)

Program P Bolivia

1. Banco Interamericano de Desarrollo, CulturaSalud/EME, REDMAS, Promundo. *Programa P Bolivia: un manual para la paternidad activa. Padres y madres por una crianza positiva, compartida y sin violencia;* Banco Interamericano de Desarrollo: Washington, D.C., United States, 2016. Available online: https://www.mencare.org/wp-content/uploads/2016/12/Programa-Bolivia-Manual-para-la-paternidad-activa.pdf (accessed on 23 April 2026)
2. Stern, E.; Alemann, C.; Delgado, G.A.F.; Vásquez, A.E. Lessons Learned from Implementing the Parenting Program P in Bolivia to Prevent Family Violence. Evaluation and Program Planning 2023, 97, 102207, https://doi.org/10.1016/j.evalprogplan.2022.102207.

Reimay – Reaching Papuan Prosperity

1. Gevers, A.; Hasyim, N. *Reimay: Reaching Papuan Prosperity. Adolescent Manual;* Partners for Prevention, UNDP, UNFPA, UN Women, UN Volunteers: January 2018. Available online: https://asiapacific.unwomen.org/sites/default/files/2025-05/reimay_reaching_papuan_prosperity_adolescent_manual_2018_comp.pdf (accessed on 23 April 2026)
2. Gevers, A.; Taylor, K. *Promising Strides toward Ending Violence against Women and Girls in the Asia and the Pacific Region: Results from Partners for Prevention Pilot Interventions in Four Countries;* Partners for Prevention, UN Women, UNFPA, UN Volunteers, UNDP: February 2018. Available online: https://prevention-collaborative.org/wp-content/uploads/2024/02/Gevers-2018-Promising-strides-towards-ending-VAWG-in-the-Asia-.pdf (accessed on 23 April 2026)
3. Moussa, J., Endut, N. *Evaluation of Partners for Prevention Regional Joint Programme for Violence against Women and Girls Prevention in Asia and the Pacific Phase II 2014 – 2017;* Partners for Prevention: April 2018. Available online: https://erc.undp.org/evaluation/documents/download/12031 (accessed on 23 April 2026)
4. Partners for Prevention. *Reimay*; Partners for Prevention: 2017.

REAL Fathers

1. Institute for Reproductive Health, Georgetown University and Save the Children. *REAL Fathers Initiative Mentor Group Discussion Guide for Less Literate Mentors;* Institute for Reproductive Health, Georgetown University: Washington, D.C., United States; Save the Children for the U.S. Agency for International Development (USAID): Washington, DC, USA, 2019. Available online: https://prevention-collaborative.org/wp-content/uploads/2021/08/REAL-Fathers_Mentor-Discussion-Guide-for-Low-Literate-Mentors-FINAL.pdf (accessed on 23 April 2026)
2. Ashburn, K.; Kerner, B.; Ojamuge, D.; Lundgren, R. Evaluation of the Responsible, Engaged, and Loving (REAL) Fathers Initiative on Physical Child Punishment and Intimate Partner Violence in Northern Uganda. *Prev Sci* **2017**, *18*, 854–864, https://doi.org/10.1007/s11121-016-0713-9.
3. Kohli, A.; Doyle, K.; Uysal, J.; Ojamuge, D.; Karamage, E.; Lundgren, R. Building a Theory of Change to Guide Fatherhood Programming to Prevent Family Violence: A Comparison of Two Programs. *BMC Public Health* **2025**, *25*, 3576, https://doi.org/10.1186/s12889-025-24883-7.

RHANI Wives

1. Raj, A.; Dasgupta, A.; Nair, S.; Battala, M.; Naik, D.D.; Silverman, J.G.; Saggurti, N.; Donta, B. *RHANI Wives HIV Risk Reduction among at risk wives in India Curriculum;* Center on Gender Equity and Health, University of California, San Diego: San Diego, California, United States; Population Council: Delhi, India; National Institute for Research in Reproductive Health: Mumbai, India, 2015.
2. Raj, A.; Saggurti, N.; Silverman, J.G.; Nair, S.; Balaiah, D. *The RHANI Wives Intervention: Combating Marital HIV Transmission;* Center on Gender Equity and Health, University of California, San Diego, California, October 2016. Available online: https://geh.ucsd.edu/wp-content/uploads/2019/04/raj-the-rhani-wives.pdf (accessed on 23 April 2026)
3. Raj, A.; Saggurti, N.; Battala, M.; Nair, S.; Dasgupta, A.; Naik, D.D.; Abramovitz, D.; Silverman, J.G.; Balaiah, D. Randomized Controlled Trial to Test the RHANI Wives HIV Intervention for Women in India at Risk for HIV from Husbands. AIDS Behav 2013, 17, 3066–3080, https://doi.org/10.1007/s10461-013-0586-x.
4. Saggurti, N.; Nair, S.; Silverman, J.G.; Naik, D.D.; Battala, M.; Dasgupta, A.; Balaiah, D.; Raj, A. Impact of the RHANI Wives Intervention on Marital Conflict and Sexual Coercion. Intl J Gynecology & Obste 2014, 126, 18–22, https://doi.org/10.1016/j.ijgo.2014.01.015.

Safe at Home

1. International Rescue Committee. *Safe at Home Women's Curriculum;* International Rescue Committee: New York, NY, United States, 2021. Available online: https://gbvresponders.org/wp-content/uploads/2022/09/SAH_Module-2_Part-3_Womens-Curriculum.pdf (accessed on 23 April 2026)
2. International Rescue Committee. *Safe at Home Men's Curriculum;* International Rescue Committee: New York, NY, United States, 2021. Available online: https://gbvresponders.org/wp-content/uploads/2022/09/SAH_Module-2_Part-3_Mens-Curriculum.pdf (accessed on 23 April 2026)
3. International Rescue Committee. *Safe at Home Family Curriculum;* International Rescue Committee: New York, NY, United States, 2021. Available online: https://gbvresponders.org/wp-content/uploads/2022/09/SAH_Module-2_Part-3_Family-Curriculum.pdf (accessed on 23 April 2026)
4. International Rescue Committee. *Safe at Home Module 1: Introduction to Safe at Home;* International Rescue Committee: New York, NY, United States, 2021. Available online: https://gbvresponders.org/wp-content/uploads/2022/09/SAH_Module-1_Introduction.pdf (accessed on 23 April 2026)
5. International Rescue Committee. *Safe at Home Module 2: Women and Children Safe at Home. Part 1: Setting Up the Safe at Home Program;* International Rescue Committee: New York, NY, United States, 2021. Available online: https://gbvresponders.org/wp-content/uploads/2022/09/SAH_Module-2_Part-1.pdf (accessed on 23 April 2026)
6. International Rescue Committee. *Safe at Home Module 2: Women and Children Safe at Home. Part 2: My Facilitation Guide;* International Rescue Committee: New York, United States, 2021. Available online: https://gbvresponders.org/wp-content/uploads/2022/09/SAH_Module-2_Part-2_-My-Facilitation-Guide.pdf (accessed on 23 April 2026)
7. International Rescue Committee. *Safe at Home Module 2: Women and Children Safe at Home. Part 5: Safe at Home Training Guide;* International Rescue Committee: New York, United States, 2021. Available online: https://gbvresponders.org/wp-content/uploads/2022/09/SAH_Module-2_Part-5_Training-Guide.pdf (accessed on 23 April 2026)
8. International Rescue Committee. *Safe at Home Module 2: Women and Children Safe at Home. Part 6: Intimate Partner Violence and Child Malreatment Co-Occurrence Guidance;* International Rescue Committee: New York, United States, 2021. Available online: https://gbvresponders.org/wp-content/uploads/2022/09/SAH_Module-2_Part-6_Co-Occurrence-Response-Guidance.pdf (accessed on 23 April 2026)
9. Falb, K.L.; Asghar, K.; Blackwell, A.; Baseme, S.; Nyanguba, M.; Roth, D.; Hategekimana, J.D.D. Improving Family Functioning and Reducing Violence in the Home in North Kivu, Democratic Republic of Congo: A Pilot Cluster-Randomised Controlled Trial of Safe at Home. BMJ Open 2023, 13, e065759, https://doi.org/10.1136/bmjopen-2022-065759.

Sammanit Jeevan

1. Volunteer Services Overseas (VSO) Nepal; South African Medical Research Council (SAMRC). *Sammanit Jeevan Living with Dignity Gender Transformative Norms Training Manual;* VSO Nepal, SAMRC, What

Works to Prevent Violence Against Women and Girls: 2013 (published 2018). Available online: https://prevention-collaborative.org/wp-content/uploads/2021/08/Zindagii-ShoistaPt1.pdf (accessed on 23 April 2026)
2. Volunteer Services Overseas (VSO) Nepal; South African Medical Research Council (SAMRC). *Sammanit Jeevan Living with Dignity Economic Empowerment and Income Generating Activities Training Manual;* VSO Nepal, SAMRC, What Works to Prevent Violence Against Women and Girls: 2013 (published 2018). Available online: https://prevention-collaborative.org/wp-content/uploads/2021/08/Zindagii-ShoistaPt2.pdf (accessed on 23 April 2026)
3. Volunteer Services Overseas (VSO) Nepal; South African Medical Research Council (SAMRC); What Works to Prevent Violence Against Women and Girls. *Evidence Brief: A family-centred intervention to prevent Violence Against Women and Girls in migrant communities of Baglung District, Nepal;* VSO Nepal, SAMRC, What Works to Prevent Violence Against Women and Girls: January 2019. Available online: https://www.aidsdatahub.org/sites/default/files/resource/family-centred-intervention-prevent-vaw-and-girls-migrant-communities-nepal-2019.pdf (accessed on 23 April 2026)
4. Shai, N.; Pradhan, G.D.; Shrestha, R.; Adhikari, A.; Chirwa, E.; Kerr-Wilson, A.; Jewkes, R. "I Got Courage from Knowing That Even a Daughter-in-Law Can Earn Her Living": Mixed Methods Evaluation of a Family-Centred Intervention to Prevent Violence against Women and Girls in Nepal. PLoS ONE 2020, 15, e0232256, https://doi.org/10.1371/journal.pone.0232256.

Samvedana Plus

1. Karnataka Health Promotion Trust (KHPT). *Reducing Intimate Partner Violence and Increasing Condom Usage in the HIV/STI Response. A Facilitators' Guide for Training Female Sex Workers;* Karnataka Health Promotion Trust: Karnataka, India, 2015. Available online: https://strive.lshtm.ac.uk/sites/strive.lshtm.ac.uk/files/Facilitator%27s%20Guide%20for%20training%20female%20sex%20workers%20Reducing%20IPV%20and%20increasing%20condom%20usage%20in%20the%20HIV%20STI%20response_0.pdf (accessed on 23 April 2026)
2. Karnataka Health Promotion Trust (KHPT). *Reducing Intimate Partner Violence and Increasing Condom Usage in the HIV/STI Response. A Facilitators' Guide for Training Intimate Partners of Female Sex Workers.* Karnataka Health Promotion Trust: Karnataka, India, 2015. Available online: https://assets.publishing.service.gov.uk/media/594a5277ed915d0baf00000a/Facilitator_s_Guide_for_training_intimate_partners_of_female_sex_workers_Reducing_intimate_partner_violence_and_increasing_condom_usage_in_the_HIV_STI_response.pdf (accessed on 23 April 2026)
3. Doddamane, M.; Raghavendra, T.; Anderson, B.; Javalkar, P.; Bhattacharjee, P. *Samvedana Plus Reducing Intimate Partner Violence Against Female Sex Workers: Implementation Design;* Karnataka Health Promotion Trust (KHPT): 2015. Available online: https://strive.lshtm.ac.uk/resources/samvedana-plus-implementation-design/ (accessed on 23 April 2026)
4. Beattie, T.S.; Isac, S.; Bhattacharjee, P.; Javalkar, P.; Davey, C.; Raghavendra, T.; Nair, S.; Ramanaik, S.; Kavitha, D.L.; Blanchard, J.F.; et al. Reducing Violence and Increasing Condom Use in the Intimate Partnerships of Female Sex Workers: Study Protocol for Samvedana Plus, a Cluster Randomised Controlled Trial in Karnataka State, South India. BMC Public Health 2016, 16, 660, https://doi.org/10.1186/s12889-016-3356-7.
5. Javalkar, P.; Platt, L.; Prakash, R.; Beattie, T.S.; Collumbien, M.; Gafos, M.; Ramanaik, S.; Davey, C.; Jewkes, R.; Watts, C.; et al. Effectiveness of a Multilevel Intervention to Reduce Violence and Increase Condom Use in Intimate Partnerships among Female Sex Workers: Cluster Randomised Controlled Trial in Karnataka, India. BMJ Glob Health 2019, 4, e001546, https://doi.org/10.1136/bmjgh-2019-001546.

SNEHA Program on Violence Against Women and Children

1. Gupta, A.D., Nevel, V. *Module on Working with Women's Group to Prevent Violence Against Women and Girls;* SNEHA Program on Prevention of Violence Against Women and Children: Mumbai, India, 2024.
2. Daruwalla, N.; Jaswal, S.; Fernandes, P.; Pinto, P.; Hate, K.; Ambavkar, G.; Kakad, B.; Gram, L.; Osrin, D. A Theory of Change for Community Interventions to Prevent Domestic Violence against Women and Girls in Mumbai, India. Wellcome Open Res. 2019, 4, 54. https://doi.org/10.12688/wellcomeopenres.15128.2

3. Daruwalla, N.; Machchhar, U.; Pantvaidya, S.; D'Souza, V.; Gram, L.; Copas, A.; Osrin, D. Community Interventions to Prevent Violence against Women and Girls in Informal Settlements in Mumbai: The SNEHA-TARA Pragmatic Cluster Randomised Controlled Trial. Trials 2019, 20, 743, https://doi.org/10.1186/s13063-019-3817-2.
4. Daruwalla, N.; Adelkar, S.; Chakraborty, P.; Copas, A.; Das, S.; Das, T.; D'Souza, V.; Gram, L.; Gupta, A.; Kakad, B.; et al. Community Interventions to Prevent Violence against Women and Girls in Informal Settlements in Mumbai: A Theory-Driven, Pragmatic, Cluster-Randomised, Controlled Trial. The Lancet Global Health 2025, 13, e2082–e2096, https://doi.org/10.1016/S2214-109X(25)00358-4.

Stepping Stones (SAMRC)

1. Jewkes, R., Nduna, M., Jama, N. *Stepping Stones A training manual for sexual and reproductive health communication and relationship skills. Edition III;* South African Medical Research Council (SAMRC): Pretoria, South Africa, 2010. Available online https://www.whatworks.co.za/resources/project-resources/item/250-stepping-stones-training-manual-for-sexual-and-reproductive-health-communication-and-relationship-skills (accessed on 23 April 2026)
2. Jewkes, R.; Wood, K.; Duvvury, N. "I Woke up after I Joined Stepping Stones": Meanings of an HIV Behavioural Intervention in Rural South African Young People's Lives. Health Education Research 2010, 25, 1074–1084, https://doi.org/10.1093/her/cyq062.
3. Jewkes, R.; Nduna, M.; Levin, J.; Jama, N.; Dunkle, K.; Puren, A.; Duvvury, N. Impact of Stepping Stones on Incidence of HIV and HSV-2 and Sexual Behaviour in Rural South Africa: Cluster Randomised Controlled Trial. BMJ 2008, 337, a506–a506, https://doi.org/10.1136/bmj.a506.

Stepping Stones, Creating Futures

1. Jewkes, R., Nduna, M., Jama, N. *Stepping Stones A training manual for sexual and reproductive health communication and relationship skills. Edition III;* South African Medical Research Council (SAMRC): Pretoria, South Africa, 2010. Available online https://www.whatworks.co.za/resources/project-resources/item/250-stepping-stones-training-manual-for-sexual-and-reproductive-health-communication-and-relationship-skills (accessed on 23 April 2026)
2. Health Economics and HIV and AIDS Research Division (HEARD), University of KwaZulu-Natal, Project Empower, and South African Medical Research Council (SAMRC). *Creating Futures: Supporting young people in building their livelihoods. A manual for facilitating livelihoods;* Health Economics and HIV and AIDS Research Division (HEARD), University of KwaZulu-Natal, Project Empower, and South African Medical Research Council (SAMRC): 2013. Available online: https://www.researchgate.net/publication/296637763_Creating_Futures_Facilitator_Manual (accessed on 23 April 2026)
3. Gibbs, A.; Washington, L.; Abdelatif, N.; Chirwa, E.; Willan, S.; Shai, N.; Sikweyiya, Y.; Mkhwanazi, S.; Ntini, N.; Jewkes, R. Stepping Stones and Creating Futures Intervention to Prevent Intimate Partner Violence Among Young People: Cluster Randomized Controlled Trial. Journal of Adolescent Health 2020, 66, 323–335, https://doi.org/10.1016/j.jadohealth.2019.10.004.
4. Gibbs, A.; Mkhwanazi, S.; Sikweyiya, Y. Stepping Stones and Creating Futures: A Group-based Approach to Addressing Violence against Women through Working with Men. J Clin Psychol 2022, 78, 26–37, https://doi.org/10.1002/jclp.23293.
5. Gibbs, A.; Jewkes, R.; Sikweyiya, Y.; Willan, S. Reconstructing Masculinity? A Qualitative Evaluation of the Stepping Stones and Creating Futures Interventions in Urban Informal Settlements in South Africa. Culture, Health & Sexuality 2015, 17, 208–222, https://doi.org/10.1080/13691058.2014.966150.
6. South African Medical Research Council (SAMRC), Project Empower, What Works to Prevention Violence Against Women and Girls. *Stepping Stones and Creating Futures: An evidence-based intervention to prevent violence against women and improve livelihoods;* SAMRC, Project Empower, What Works to Prevention Violence Against Women and Girls: October 2018. Available online: https://whatworks.co.za/documents/publications/242-stepping-stones-web/file (accessed on 23 April 2026)

Stopping Abuse and Female Exploitation (SAFE)

1. ECORYS; Social Development Direct. *Toose Implementation Guide: A step-by-step guide to provide information and tools for how to implement Toose;* Stopping Abuse and Female Exploitation (SAFE) Programme: 2019. Available online: https://www.ecorys.com/app/uploads/2019/02/1.-Toose-Implementation-Guide.pdf (accessed on 23 April 2026)
2. ECORYS; Social Development Direct. *Toose Social Empowerment Facilitator's Guide. A guide for facilitators to follow as they take groups through the social empowerment component of the Toose model;* Stopping Abuse and Female Exploitation (SAFE) Programme: 2019. Available online: https://www.ecorys.com/app/uploads/2019/02/3.-Toose-Social-Empowerment-Facilitators-Guide.pdf (accessed on 23 April 2026)
3. ECORYS; Social Development Direct. *Toose Integrating GBV Response and Handling Disclosures During Toose Sessions. A Guide for Facilitators;* Stopping Abuse and Female Exploitation (SAFE) Programme: 2019. Available online: https://www.ecorys.com/app/uploads/2019/02/4.-Toose-Response-Guide.pdf (accessed on 23 April 2026)
4. ECORYS; Social Development Direct. *Toose Fidelity Brief for Adapting Toose. A practical guide on what it means to have 'fidelity' to the Toose intervention;* Stopping Abuse and Female Exploitation (SAFE) Programme: 2019.
5. ECORYS; Social Development Direct; SafAIDs; TetraTech. *SAFE Learning Brief: What Works in GBV Prevention in Zimbabwe?* Stopping Abuse and Female Exploitation (SAFE) Programme: 2025. Available online: https://intdev.tetratecheurope.com/wp-content/uploads/2025/04/SAFE-Learning-Brief-2025.pdf (accessed on 23 April 2026)
6. ECORYS; Social Development Direct; SafAIDs; TetraTech. *SAFE Impact Brief: Evidence and Impact from GBV prevention in Zimbabwe.* Stopping Abuse and Female Exploitation (SAFE) Programme: 2025. Available online: https://intdev.tetratecheurope.com/wp-content/uploads/2025/04/SAFE-Impact-Brief-2025.pdf (accessed on 23 April 2026)

Sumaq Warmi

1. Inter-American Development Bank. *Manual de Capacitación sobre Género y Violencia.* Inter-American Development Bank: n.d.
2. Agüero, J.M.; Frisancho, V. *Sumaq Warmi: Reducing Violence Against Women in Microfinance*; Inter-American Development Bank: February 2018. Available online: https://publications.iadb.org/en/sumaq-warmi-reducing-violence-against-women-microfinance (accessed on 23 April 2026)

Together Ending Violence Against Women (TEVAW)

1. Bantwana World Education Initiative. *Male Peer Groups: A Facilitator's Guide;* Bantwana World Education Initiative; South African Medical Research Council (SAMRC): 2018. Available online: https://bantwana.org/wp-content/uploads/2018/01/TEVAW-Male-Peer-Groups-Facilitator-Guide.pdf (accessed on 23 April 2026)
2. Bantwana World Education Initiative, Boston University, Sexual Violence Research Initiative (SVRI).*Together To End Violence Against Women Technical Brief.* Bantwana World Education Initiative, Boston University, Sexual Violence Research Initiative (SVRI): 2018. Available online: https://bantwana.org/resource/tevaw-tech-brief/ (accessed on 23 April 2026)
3. Halim, N.; Steven Mzilangwe, E.; Reich, N.; Badi, L.; Simmons, E.; Servidone, M.; Bingham Holmes, N.; Kawemama, P.; Messersmith, L.J. Together to End Violence against Women in Tanzania: Results of a Pilot Cluster Randomized Controlled Trial to Evaluate Preliminary Effectiveness of Interpersonal and Community Level Interventions to Address Intimate Partner Violence. Global Public Health 2019, 14, 1653–1668, https://doi.org/10.1080/17441692.2019.1609062.

Unite for a Better Life

1. Rolleri, L., Verani, F., Tewolde, S. *A Gender Transformative Intimate Partner Violence and HIV Prevention Curriculum for Couples;* Abdul Latif Jameel Poverty Action Lab (J-PAL) at Massachusetts Institute of Technology, EngenderHealth, Ethiopian Public Health Association, 2014.

2. Rolleri, L., Verani, F., Tewolde, S. *Unite for a Better Life for Men. A Gender Transformative Intimate Partner Violence and HIV Prevention Curriculum for Men;* Abdul Latif Jameel Poverty Action Lab (J-PAL) at Massachusetts Institute of Technology, EngenderHealth, Ethiopian Public Health Association, 2014.
3. Rolleri, L., Verani, F., Tewolde, S. *Unite for a Better Life for Women. A Gender Transformative Intimate Partner Violence and HIV Prevention Curriculum for Women;* Abdul Latif Jameel Poverty Action Lab (J-PAL) at Massachusetts Institute of Technology, EngenderHealth, Ethiopian Public Health Association, 2014.
4. Salgado, I.; Leight, J.; Sharmva, V. *Preventing Intimate Partner Violence by Engaging Men: Evidence from Unite for Better Life in Ethiopia*. Poverty action lab, 2 December 2020. Available online: https://www.povertyactionlab.org/blog/12-2-20/preventing-intimate-partner-violence-engaging-men-evidence-unite-better-life-ethiopia (accessed on 22 April 2026)
5. Sharma, V.; Leight, J.; Verani, F.; Tewolde, S.; Deyessa, N. Effectiveness of a Culturally Appropriate Intervention to Prevent Intimate Partner Violence and HIV Transmission among Men, Women, and Couples in Rural Ethiopia: Findings from a Cluster-Randomized Controlled Trial. PLoS Med 2020, 17, e1003274, https://doi.org/10.1371/journal.pmed.1003274.

Vietnam Male Advocate Clubs

1. Gevers, A.; Trang, Q.T.; Phuong, L.T.L.; An, P.T. *A Manual for Facilitators of Male Advocate Clubs to Prevent Violence Against Women and Girls. Tranforming masculinities and building respectful and equal relationships with women;* Da Nang Women's Union, Partners for Prevention: Da Nang, Viet Nam, November 2016. Available online: https://asiapacific.unwomen.org/en/digital-library/publications/2017/04/the-manual-for-facilitators-of-male-advocate (accessed on 23 April 2026)
2. Gevers, A.; Taylor, K. *Promising Strides toward Ending Violence against Women and Girls in the Asia and the Pacific Region: Results from Partners for Prevention Pilot Interventions in Four Countries.* Partners for Prevention, UN Women, UNFPA, UN Volunteers, UNDP: February 2018. Available online: https://prevention-collaborative.org/wp-content/uploads/2024/02/Gevers-2018-Promising-strides-towards-ending-VAWG-in-the-Asia-.pdf (accessed on 23 April 2026)
3. Moussa, J., Endut, N. *Evaluation of Partners for Prevention Regional Joint Programme for Violence against Women and Girls Prevention in Asia and the Pacific Phase II 2014 – 2017;* Partners for Prevention: April 2018. Available online: https://erc.undp.org/evaluation/documents/download/12031 (accessed on 23 April 2026)
4. Partners for Prevention. *Engaging Men and Boys to Prevent Gender-Based Violence: Good practices and Lessons Learned from Male Advocate Clubs in Da Nang.* Partners for Prevention: 2017. Available online: https://asiapacific.unwomen.org/sites/default/files/2025-05/engaging-men-and-boys-to-prevent-gbv-good-practices-and-lessons-learned-from-male-advocate-clubs-da-nang-2017.pdf (accessed on 23 April 2026)
5. Partners for Prevention. *Lessons Learned Report Male Advocate Club Project in Da Nang, Viet Nam;* Partners for Prevention: 2017
6. Nguyen, H.T.; Tran, P.K.; Gevers, A.; Taylor, K. *A Qualitative Endline Study of a Male Advocate Intervention to Prevent Violence against Women and Girls in Da Nang, Viet Nam.* Da Nang City, UN Women, Partners for Prevention: 2017. Available online: https://asiapacific.unwomen.org/sites/default/files/2025-05/a-qualitative-endline-study-of-a-male-advocate-intervention-to-prevent-vawg-in-da-nang-viet-nam-2017.pdf (accessed on 23 April 2026)
7. Partners for Prevention. *Male Advocate Programme;* Partners for Prevention: 2017

Wanawake Na Maisha

1. Rolleri, L., Verani, F., Lees, S., Nyalali, K., Kiswaga, L. *Wanawake Na Maisha: A Gender Transformative Intimate Partner Violence Prevention Curriculum for Women in Mwanza, Tanzania;* London School of Hygience and Tropical Medicine, London, EngenderHealth, New York, Mwanza Intervention Trials Unit at the National Institute for Medical Research, Mwanza, Tanzania: 2014. Available online: https://prevention-collaborative.org/wp-content/uploads/2021/08/Maisha-Wanawake-Na-Maisha_FINAL_Dec2015.pdf (accessed on 23 April 2026)
2. Harvey, S.; Abramsky, T.; Mshana, G.; Hansen, C.H.; Mtolela, G.J.; Madaha, F.; Hashim, R.; Kapinga, I.; Watts, C.; Lees, S.; et al. A Cluster Randomised Controlled Trial to Evaluate the Impact of a Gender Transformative Intervention on Intimate Partner Violence against Women in Newly Formed

Neighbourhood Groups in Tanzania. BMJ Glob Health 2021, 6, e004555, https://doi.org/10.1136/bmjgh-2020-004555.
3. Kapiga, S.; Harvey, S.; Mshana, G.; Hansen, C.H.; Mtolela, G.J.; Madaha, F.; Hashim, R.; Kapinga, I.; Mosha, N.; Abramsky, T.; et al. A Social Empowerment Intervention to Prevent Intimate Partner Violence against Women in a Microfinance Scheme in Tanzania: Findings from the MAISHA Cluster Randomised Controlled Trial. The Lancet Global Health 2019, 7, e1423–e1434, https://doi.org/10.1016/S2214-109X(19)30316-X.
4. McLean, L.; Jayasekara, P.; Stern, E. *Programme Summary: Maisha Intervention in Tanzania.* Prevention Collaborative: 2020.

Walter Reed Program/Henry Jackson Foundation (WRP/HJFMRI) GBV Program

1. EngenderHealth; CHAMPION Project Tanzania. *CoupleConnect: A Gender-Transformative HIV Prevention Curriculum for Tanzanian Couples*; EngenderHealth: Dar es Salaam, Tanzania, 2014.
2. Settergren, S. Tathmini GBV: Evaluation of the Impact of Comprehensive Gender-Based Violence Programming in Mbeya, Tanzania. Presented at the SVRI Forum, Stellenbosch, South Africa, 16 September 2015.
3. Palladium; Project SOAR. Tathmini GBV Study: Evaluation of Comprehensive GBV Programming Delivered through the HIV Program Platform in Tanzania. Presented at the Project SOAR Technical Advisory Network Meeting: Tanzania, 2017.
4. EngenderHealth; RESPOND Tanzania Project. *RESPOND Tanzania Project Quarterly Report: April 1, 2015–June 30, 2015*; EngenderHealth: Dar es Salaam, Tanzania, 2015.
5. E2A Project. *Couple-Focused Interventions: A Theory of Change*; E2A Project: Washington, DC, USA, 2021. Available online: https://www.pathfinder.org/wp-content/uploads/2024/01/Global-Couple-Focused-Interventions-TOC-ENG.pdf (accessed on 23 April 2026)

Yaari Dosti

1. Population Council; CORO for Literacy; MAMTA; Instituto Promundo. Yaari Dosti: Young Men Redefine Masculinity; Population Council, New Delhi, CORO for Literacy, Mumbai, MAMTA, New Delhi, Instituto Promundo, Rio de Janeiro, Brazil, 2006. Available online: https://knowledgecommons.popcouncil.org/cgi/viewcontent.cgi?article=1593&context=departments_sbsr-hiv (accessed on 23 April 2026)
2. Verma, R.; Pulerwitz, J.; Mahendra, V.; Khandekar, S.; Singh, A.K.; Das, S.S.; Mehra, S.; Nura, A.; Barker, G. *Promoting Gender Equity as a Strategy to Reduce HIV Risk and Gender-Based Violence among Young Men in India*; Population Council: 2008. Available online: https://knowledgecommons.popcouncil.org/cgi/viewcontent.cgi?article=1030&context=departments_sbsr-hiv (accessed on 23 April 2026)
3. Verma, R.K.; Pulerwitz, J.; Mahendra, V.; Khandekar, S.; Barker, G.; Fulpagare, P.; Singh, S.K. Challenging and Changing Gender Attitudes among Young Men in Mumbai, India. Reproductive Health Matters 2006, 14, 135–143.
4. Doyle, K.; Kato-Wallace, J. *Program H: A review of the evidence: Nearly two decades of engaging young men and boys in gender equality*; Equimundo: Washington, D.C., United States, 2021. Available online: https://www.equimundo.org/resources/program-h-a-review-of-the-evidence-nearly-two-decades-of-engaging-young-men-and-boys-in-gender-equality/ (accessed on 23 April 2026)

Zindagii Shoista

1. Jewkes, R., Jama-Shai, N. *Zindagii Shoista Living with Dignity: Workshop Manual. Part 1: Social empowerment;* What Works to Prevent Violence Against Women and Girls, South African Medical Research Council, Cesvi, International Alert: 2018. Available online: https://www.whatworks.co.za/documents/publications/curricula/221-tajikistan-livingwithdignity-pt1-en-2018-final/file (accessed on 23 April 2026)
2. Gulyamova, P.; Balboni, S.; Jewkes, R.; Jama-Shai, N. *Zindagii Shoista Living with Dignity: Workshop Manual. Part 2: Enabling economic empowerment through income generating activities.* What Works to Prevent Violence Against Women and Girls, South African Medical Research Council, Cesvi,

International Alert: 2018. Available online: https://www.whatworks.co.za/resources/item/540-zindagii-shoista-living-with-dignity-workshop-manual-part-2 (accessed on 23 April 2026)
3. Mastonshoeva, S.; Shonasimova, S.; Gulyamova, P.; Jewkes, R.; Shai, N.; Chirwa, E.; Myrttinen, H. Quantitative Evaluation of Zindagii Shoista (Living with Dignity) Intervention to Prevent Violence against Women in Tajikistan. Global Health Action 2022, 15, 2122994, https://doi.org/10.1080/16549716.2022.2122994.
4. Mastonshoeva, S.; Myrttinen, H.; Chirwa, E.; Shonasimova, S.; Gulyamova, P.; Shai, N.; Jewkes, R. *Evaluation of Zindagii Shoista (Living with Dignity), an Intervention to Prevent Violence against Women in Tajikistan: Impact after 30 Months;* What Works to Prevent Violence Against Women and Girls: 2020. Available online: https://www.whatworks.co.za/resources/reports/item/687-evaluation-of-zindagii-shoista-living-with-dignity-an-intervention-to-prevent-violence-against-women-in-tajikistan-impact-after-30-months (accessed on 23 April 2026)
5. Mastonshoeva, S.; Shonasimova, S.; Gulyamova, P.; Jewkes, R.; Shai, N.; Myrttinen, H. *Zindagii Shoista Living with Dignity: Mixed-methods evaluation of intervention to prevent violence against women in Tajikistan;* What Works to Prevent Violence Against Women and Girls/SAMRC: 2019**.** Available online: https://www.international-alert.org/app/uploads/2021/08/Tajikistan_LivingWithDignity_Evaluation_EN_2019.pdf (accessed on 23 April 2026)
6. UN Women; Social Development Direct. *RESPECT Preventing Violence against Women Programme Summary: Zindagii Shoista, Tajikistan;* UN Women/Social Development Direct: 2020. Available online: https://www.unwomen.org/sites/default/files/Headquarters/Attachments/Sections/Library/Publications/2020/RESPECT-implementation-guide-Programme-summary-Zindagii-shoista-en.pdf (accessed on 23 April 2026)
