# Supplementary material for: A Scoping Review of IPV Prevention Curricula and Their Implementation in Low- and Middle-Income Countries: Implications for the Next Generation of Programs
Source: Int J Environ Res Public Health. 2026 Jul 29;23(8):989. doi: 10.3390/ijerph23080989 (PMC13512808; doi:10.3390/ijerph23080989)
Supplement: Supplementary file 1 [file ijerph-23-00989-s001.zip › Table S1 Overview of Programs (n=42) and Curricula (n=55).pdf]

Supplemental Table S1. Overview of programs (n=42) and curricula (n=55)

#	Program	Country (setting)	Partners, years	Curriculum	Curriculum Population/Participants	Session intensity and frequency[a]	Facilitators[b]
1	**Bandebereho** – "role model"	Rwanda (rural)	RWAMREC, Equimundo (2013-2015)	Bandebereho Father's manual [87]	**Couples:** Expectant or current fathers (child under 5 years) ages 21 to 35 years, and their partners	15 weekly sessions: 7 for men and 8 for couples (60 hours)	Peers (male)
				Bandebereho Young men's manual [88]	**Adolescent boys and young men**: ages 18 to 24 years, unmarried	15 weekly sessions (60 hours)	Peers (male)
				Bandebereho Young women's manual [89]	**Adolescent girls and young women**: ages 18 to 24 years, unmarried	15 weekly sessions (60 hours)	Peers (female)
2	**Becoming One**	Uganda (rural)	World Vision, IRC Airbel Lab (2017-2019)	Faith Leaders Manual [41]	**Couples:** ages 18 to 65 years, living together for at least one year	12 weekly sessions (18 hours)	Community (faith) leaders (female or male)
3	**Change Starts at Home**	Nepal (rural)	Equal Access International (2015-2017)	BIG Change Curriculum for listening and discussion group facilitators [90]	**Couples:** Married women who are 18 to 48 years, who reside most of the year with their husbands, and their partners	40 weekly sessions (80 hours), includes sessions for men and women together & separately	Community leaders (female or male)
4	**Community-Based Prevention of Violence against Refugee Women and Girls**	Kenya (urban or peri-urban)	HIAS Refugee Trust of Kenya (2019-2022)	Men's group discussion guide [91]	**Men:** Male partners of female refugees in Nairobi country	20 sessions, once or twice per week (40 hours)	Community leaders (male)
5	**Do Kadam Barabari Ki Ore** – "Two steps towards equality"	India (rural)	Population Council, Centre for Catalyzing Change (C3), London School of Hygiene and Tropical Medicine (2012-2016)	Peer Mentors Module for SHG Women and Husband Peer Mentors (women's sessions) [92]	**Women** (couples): 18 to 49 years, in existing self-help groups	22 sessions, once every two weeks to once a month (44 hours)	Peers (female)
				Peer Mentors Module for SHG Women and Husband Peer Mentors (husband's sessions) [92]	**Men** (couples): male partners of women in existing self-help groups (linked to above)	12 monthly sessions (24 hours)	NGO staff or field agents (male)
				Gender transformative life skills education curriculum for young men [93]	**Adolescent boys and young men:** ages 13 to 21 years, unmarried, from existing youth clubs	42 weekly sessions (96 hours)	Peers and NGO staff (male)

6	**EA$E**	Cote d'Ivoire (rural)	International Refugee Center (IRC) (2007-2010)	EA$E Discussion series facilitator's guide [94]	**Couples:** Women 18 years and older recruited to VSLAs, and their male partners	8 sessions, delivered every two weeks (16 hours)	NGO staff or field agents (sex unclear)
7	**Engaging Men in Accountable Practice** (EMAP)	Democratic Republic of Congo (DRC), (conflict-affected/ humanitarian)	IRC (2016-2017)	Men's Curriculum Engaging Men in Accountable Practice [95]<br><br>Women's Curriculum Engaging Men in Accountable Practice [95]	**Men:** ages 18 and older, living stably in the community<br><br>**Women:** open to all women in the community ages 20 and older, living stably in the community	16 weekly sessions (48 hours)<br><br>8 weekly sessions (16 hours)	NGO staff or field agents (male)<br><br>NGO staff or field agents (female)
8	**Engaging with Faith Groups to Prevent Violence Against Women and Girls in Conflict-Affected Communities**	DRC (conflict-affected/ humanitarian)	Tearfund, HEAL Africa (2015-2018)	Transforming Masculinities Community Dialogues Facilitator Manual [96]	**Men and women:** ages 18 to 75 years, affiliated to local faith communities	6 weekly sessions (12 hours); includes sessions for men and women separately (5) & together (1)	Community leaders (female and male)
9	**Gender Responsive School Model Pilot Project**	Vietnam (urban)	Plan International Vietnam, Department of Education and Training, Cetner for Study and Applied Science on Children, Women and Adolescents (CSAGA), ICRW (2013-2016)	Training Manual on School Related Gender Based Violence Prevention and Response for Teachers of lower secondary schools [97]<br><br>Training Manual on School Related Gender Based Violence Prevention and Response for Teachers of high schools [98]	**Adolescent boys and girls:** Lower secondary school students (grade 6-8), who participate as part of school's extracurricular activities<br><br>**Adolescent boys and girls:** Upper secondary school students (grade 10-11), who participate as part of school's extracurricular activities	24 monthly sessions (18 hours)<br><br>17 monthly sessions (13 hours)	Teachers (female or male)<br><br>Teachers (female or male)
10	**Get Into Rugby Plus**	Fiji (urban and rural)	Oceania Rugby, UN Women, Childfund Rugby (2017-present)	Get Into Rugby Plus Curriculum [99]	**Adolescent boys and girls:** ages 11 to 14 years	16 sessions, frequency unclear (13 hours)	Teachers (rugby coaches) (female or male)
11	**Ghya Bharari Ekatra –** "Fly High Together"	India (urban or peri-urban)	IMR-NARI (2017-2018)	Flying High Together Curriculum [48]	**Adult couples:** newly married couples in urban slum communities	6 weekly sessions (12 hours); includes sessions for men and women separately (1) and together (5)	Peers (female and male)
12	**HERrespect**	India (urban or peri-urban)	Business for Social Responsibility (BSR), South African Medical Research Council	Female factory workers curriculum [100]	**Women:** working in urban/semi-urban factories	6 sessions, delivered weekly to monthly (18 hours)	NGO staff or field agents (sex unknown)

		(SAMRC), icddr,b, Change Associates Limited, WE CAN (2015-2018)	Male factory workers curriculum [101]<br><br>Factory management curriculum [102]	**Men:** working in urban/semi-urban factories<br><br>**Men and women:** managers in urban/semi-urban factories	6 sessions, delivered weekly to monthly (18 hours)<br>6 sessions, delivered weekly to monthly (18 hours)	NGO staff or field agents (sex unknown)<br>NGO staff or field agents (sex unknown)
13	**Humqadam –** "Companion who walks alongside"	Pakistan (semi-urban)	Rozan (2009-2011)	Engaging with boys and young men to address gender-based violence and masculinities [103]	**Adolescent boys and young men:** ages 16 to 30 years	15 sessions, delivered weekly or every two weeks (32 hours)	Male facilitators (type unknown)
14	**Intervention with Microenterprise for Aids and Gender Equity (IMAGE) Study**	South Africa (rural)	Rural AIDS & Development Action Research Program (RADAR), University of Witswatersrand School of Public Health, Small Enterprise Foundation (2001-2004)	Sisters for Life curriculum [49]	**Women:** Poorest women (ages 18+) in rural areas who are enrolled in a microfinance program	10 sessions, delivered every two weeks (11 hours)	NGO staff or field agents (sex unknown)
15	**Improved Action Against Sexual and Gender-Based Violence and Harmful Practices**	Nigeria (rural)	Scripture Union West Africa (SUWA) (2020-2022)	Community dialogues facilitators manual on sexual and gender-based violence [104]	**Men and women:** from the local community	10 weekly sessions (20 hours); sessions for men and women separately (9) and together (1)	Community leaders (female and male)
16	**Indashyikirwa** – "Agents of Change"	Rwanda (rural)	CARE Rwanda, RWAMREC, Rwanda Women's Network (2014-2018)	Indashyikirwa couples curriculum [105]	**Couples:** Women in village savings and loans associations (VSLAs) and their male partners	21 weekly sessions (60 hours)	NGO staff or field agents (female and male)
17	**Managing Work and Life**	Ethiopia (urban and rural)	CMI, Hiwot Ethiopia (2018-2019)	A gender transformative intimate partner violence prevention course for women in Ethiopia [42]	**Women:** ages 18 to 30 years, married or living with a partner	10 weekly sessions (20 hours)	Teachers (female)
18	**Masculinité, Famille, et Foi** – "Masculinity, Family and Faith"	DRC (urban)	Tearfund, Population Services International (PSI), Association de Santé Familiale (ASF), Institute for Reproductive Health (IRH) at Georgetown University (2015-2018)	Community dialogues: promoting respectful relationships and equitable communities – with sessions on child spacing [106]	**Couples:** Women ages 18 to 35 years and their male partners, newly married or first-time parents	9 weekly sessions (17 hours); sessions for men and women separately (5) and together (4)	Community leaders (female and male)

19	**Men in Partnership**	Cote d'Ivoire (conflict-affected/ humanitarian)	IRC (2010-2012)	Men and women in partnership facilitator guide [107]	**Adolescent boys and adult men:** Any male community member age 15 years and older	16 weekly sessions (46 hours)	NGO staff or field agents (male)
20	**I Can Say No**	Serbia (urban or peri-urban)	Autonomous Women's Center, Fundacion Privada Indera, Centar Za edukaciju Savjetovanje I Istrazivanje (CESI), Nok a Nokert Egyutt Az Eroszak Ellen Egyesulet (NANE) (2019-2021)	Capacity Training Manual to raise awareness and contribute to ending Cyber sexual and gender-based violence in youth intimate partner relationships [108]	**Adolescent boys and girls:** ages 14-19 years in secondary schools	8 sessions, most often delivered through a two-day intensive workshop (14 hours)	Peers (female)
21	**Parenting for Respectability**	Uganda (rural)	Makerere University (Child Health and Development Center) (2013-2022)	Parenting for Respectability Curriculum [109]	**Men and women:** parents of children 0 to 17 years (attendees can, but do not have to participate with their partner)	16 weekly sessions (32 hours); sessions for men and women separately (9) and together (7)	Peers (female and male)
22	**Planim Save, Kamap Strongpela** (PSKS) – "Plant Knowledge, Grow Strong"	Papua New Guinea (urban)	Nazareth Centre for Rehabilitation, UN Women, UNICEF (2014-2016)	Planim Save, Kamap Strongpela curriculum: a guide for promoting community social cohesion and security in Bougainville [50]	**Men and women:** community members ages 18 to 83 years old	22 sessions (44 hours), frequency unknown	Community leaders (female and male)
23	**Program Abb**	Lebanon (urban and rural)	Abaad, Equimundo, Ministry of Social Affairs (2017-2019)	Program Abb – Early Childhood Development [22]	**Couples:** Men who are parents of children 0 to 3 years and their partners, including Lebanese and Syrian refugee families	13 sessions, delivered weekly or once every two weeks (18 hours); sessions for men (8) and for couples (5)	NGO staff (female or male)
24	**Program P Bolivia**	Bolivia (urban)	Inter-American Development Bank, Consejo de Salud Rural Andino (2015-2017)	Programa P Bolivia: un manual para la paternidad activa – sesiones con papás [110]	**Men** (couples): Indigenous father of a child 0 to 3 years, cohabiting with partner	10 sessions, delivered weekly or every two weeks (20 hours)	Male NGO staff or field agents (male)
				Programa P Bolivia: un manual para la paternidad activa – sesiones con mamás [110]	**Women** (couples): Indigenous mother of a child 0 to 3 years, cohabiting with partner (linked to above)	9 sessions, delivered weekly or every two weeks, (15 hours)	NGO staff or field agents (female)
25	**Reimay – Reaching Papuan Prosperity**	Indonesia (semi-rural)	Partners for Prevention, UNFPA, UN Women and UNV, Indonesian Family Planning Association (PKBI)	Reimay: Reaching Papuan Prosperity Adolescent Manual [111]	**Adolescent boys and girls:** ages 12 to 16 years in junior high school	22 sessions, delivered every two weeks (77 hours)	NGO staff or field agents (female and male)

			Jayapura and Lembaga Pegkajian Pemberdayaan Perempuan dan Anak (Lp3A) Jayapur (2015-2017)				
26	**Responsible, Engaged, and Loving (REAL) Fathers**	Uganda (conflict-affected/ humanitarian)	Save the Children, IRH (2013-2015)	REAL Fathers mentor group discussion guide for less literate mentors [51]	**Couples:** Fathers ages 16 to 25 years, with children ages 0 to 3, and their partners	12 sessions, delivered every two weeks (18 hours); includes individual mentoring (6) & group sessions (6); women participate in 2 individual and 1 group session	Community leaders (male)
27	**RHANI Wives**	India (urban)	University of California San Diego, Population Council, National Institute for Research in Reproductive Health (2009-2011)	RHANI Wives Curriculum [112]	**Women:** Married women 18 to 40 years who reported violence and/or heavy drinking by partner	6 sessions, delivered weekly or every two weeks (6 hours); includes 4 individual and 2 group sessions	Counselors (sex unknown);
28	**Safe at Home**	DRC (conflict-affected/ humanitarian)	IRC (2017-2021)	Safe at Home Women's Curriculum [113] + Safe at Home Family Curriculum [114]	**Women** (couples): ages 20 to 50 years old, with a child 6 to 12 years	23 weekly sessions (51 hours); includes sessions for women (18) & family (5)	Community members (female)
				Safe at Home Men's Curriculum [115]+ Safe at Home Family Curriculum [114]	**Men** (couples): ages 20 to 50 years old, with a child 6 to 12 years (linked to above)	29 weekly sessions (87 hours); includes sessions for men (24) & family (5)	Community members (male)
29	**Sammanit Jeevan** – Living with Dignity	Nepal (rural)	VSO Nepal, SAMRC (2016-2018)	Sammanit Jeevan: Gender transformative norms training manual [116] + Sammanit Jeevan: Economic Empowerment and Income Generating Activities Training Manual [117]	**Men and women:** Young married women, their husbands and cohabiting mothers-and fathers-in-law (only women participate in all sessions)	20 weekly sessions (60 hours); 13 social empowerment sessions and 7 economic empowerment sessions; 9 single-sex sessions and 11 mixed-sex	Peers (female and male)
30	**Samvedana Plus**	India (urban)	Karnataka Health Promotion Trust (KHPT), Chaitanya AIDS Tadegattuva Mahila Sangha (2015-2017)	Reducing Intimate Partner Violence and Increasing Condom Usage in the HIV/STI Response: A facilitators guide for training female sex workers [118]	**Women** (couples): Female sex workers	12 weekly sessions (24 hours)	Community leaders (female)
				Reducing Intimate Partner Violence and Increasing Condom Usage in the HIV/STI Response: A facilitators guide	**Men** (couples): Male partners of female sex workers (linked to above)	6 monthly sessions (13 hours)	Community leaders (male)

				for training intimate partners of female sex workers [119]			
31	**SNEHA Program on Violence Against Women and Children (Taking Action, Reaching All (TARA) trial)**	India (urban or peri-urban)	Society for Nutrition, Education and Health Action (SNEHA), University College London (2018-2023)	Women's group module [120]	**Women:** Adult women in urban slums	60 sessions, delivered every two weeks (75 hours)	Community leaders (female)
32	**Stepping Stones** (SAMRC)	South Africa (rural)	SAMRC, Salamander Trust (2004-2006)	Stepping Stones Version III (SAMRC Version) [43]	**Female and male adolescents and young adults**: ages 16 to 23 years living in the community	13 sessions delivered twice weekly (50 hours); sessions for men and women separately (10) and together (3)	NGO staff or field agents (male and female)
33	**Stepping Stones, Creating Futures**	South Africa (urban)	Project Empower, SAMRC (2015-2018)	Stepping Stones Version III (SAMRC Version) [43] + Creating Futures manual [121]	**Young men and women**: ages 18 to 30 living in informal settlements (not in relationships with each other)	21 sessions delivered twice weekly (63 hours); includes social empowerment sessions (10) and economic empowerment sessions (11); all sessions single-sex	Peers (female and male)
34	**Stopping Abuse and Female Exploitation (SAFE)**	Zimbabwe (urban and rural)	Ecorys, Social Development Direct, Plan INtenrational Zimbabwe, Caritas Harare, Mwenezi Development Training Centre, Musasa (2019-2024)	Toose Social Empowerment Facilitators Guide [122]	**Couples:** engaged in livelihoods (savings groups) or social protect programming (cash transfers), aged 18 years and older	10 sessions, delivered weekly or every two weeks (28 hours)	Peers and NGO staff (female and male)
35	**Sumaq Warmi** – "Wonderful Woman"	Peru (rural)	IDB (2014-2016)	Manual de Capacitación sobre Género y Violencia [123]	**Women:** ages 18 and above who were existing clients in a microfinance (village banking) program	7 monthly sessions (14 hours)	NGO staff (female)
36	**Together Ending Violence against Women** (TEVAW)	Tanzania (rural)	World Education Inc (WEI)/Bantwana, Boston University Center for Global Health and Development (2015-2016)	Male peer Groups Facilitators Guide [124]	**Men:** Male partners of women participating in the LIMCA livelihoods program	25 sessions delivered through a 4-day intensive workshop (24 hours)	NGO staff (male)

37	**Unite for a Better Life (UBL)**	Ethiopia (rural)	J-PAL, EngenderHealth, Addis Ababa University, Ethiopian Public Health Association (2014-2018)	Unite for a Better Life Women Curriculum [125]	**Women** (couples): Women ages 18 to 49 who are married or cohabiting with a partner	14 sessions delivered twice weekly (38 hours)	Community leaders (female)
				Unite for a Better Life Men Curriculum [126]	**Men** (couples): Male partners of women who are 18 to 49 (linked to above)	14 sessions delivered twice weekly (38 hours)	Community leaders (male)
				Unite for a Better Life Couples Curriculum [127]	**Couples:** Women ages 18 to 49 and their male partners	14 sessions delivered twice weekly (38 hours)	Community leaders (female and male)
38	**Vietnam Male Advocate Clubs**	Vietnam (urban or peri-urban)	Partners for Prevention, UN Women, UNV, Da Nang Women's Union (2015-2016)	A Manual for Facilitators of Male Advocate Clubs to Prevent Violence against Women and Girls [128]	**Young and adult men:** Men ages 18 to 60 years, including students, young professionals, and community leaders	16 sessions delivered every two weeks and then monthly (24 hours)	NGO staff or field agents (male)
39	**Wanawake Na Maisha** – Women and Life	Tanzania (urban)	Engender Health, MITU, LSHTM (2014-2019)	Wanawake Na Maisha curriculum [129]	**Women:** of low socioeconomic status recruited into microfinance groups	10 sessions delivered every two weeks (20 hours)	NGO staff or field agents (female)
40	**Walter Reed Program/Henry Jackson Foundation (WRP/HJFMRI) GBV Program**	Tanzania (urban)	Engender Health, WRP/HJFMRI, PEPAR, US Department of Defense (2012-2016)	Couple Connect Curriculum [130]	**Couples:** Married/cohabiting nonpolygamous couples, aged 20 and older, in a relationship 10 years or less, literate	15 weekly sessions (36 hours)	Peers (female and male)
41	**Yaari Dosti** – "Bonding among men"	India (urban and rural)	CORO for Literacy, MAMTA, Population Council, Instituto Promundo (2005-2006)	Yaari Dosti: Young men redefine masculinity [131]	**Adolescent boys and young men:** 16 to 24 years in low-income communities, recruited primarily from existing vocational, political, religious or cultural groups	20 weekly sessions (20 hours)	Peers (male)
42	**Zindagii Shoista** – "Living with Dignity"	Tajikistan (rural)	International Alert, CESVI, SAMRC (2015-2018)	Zindagii Shoista Workshop Manual: Part 1 Social Empowerment [132] + Part 2: Enabling Economic Empowerment through Income-Generating Activities [133]	**Men and women:** Young women, older women, young men, older men within the same extended families (only women participate in all sessions)	23 weekly sessions (60 hours); includes separate social (11) and economic empowerment sessions (12)	NGO staff or field agents (female and male)

a Total instruction time has been rounded to the closest hour for the purposes of the table; the exact time in hours and minutes was used for analysis in the main text.
b Facilitators were assigned into five broad categories: **NGO staff or field agents**, which includes gender specialists or individuals with facilitation experience who are hired by an organization to deliver sessions; **peers**, which includes individuals of similar background characteristics as participants, sometimes elected or selected from within the group; **community leaders**, which included respected community members, faith leaders, and elected leaders; **teachers**, who might be delivering programs to the students in their classroom or hired to deliver programming in the community; and **service providers**, which included health or other frontline workers.
