# Supplementary material for: A Scoping Review of IPV Prevention Curricula and Their Implementation in Low- and Middle-Income Countries: Implications for the Next Generation of Programs
Source: Int J Environ Res Public Health. 2026 Jul 29;23(8):989. doi: 10.3390/ijerph23080989 (PMC13512808; doi:10.3390/ijerph23080989)
Supplement: Supplementary file 1 [file ijerph-23-00989-s001.zip › Table S2 Definitions of key topic areas in IPV prevention curricula.pdf]

Supplemental Table S2. Definition of key topic areas in IPV prevention curricula

Topic Area	Description of subject matter
**Understanding and addressing violence**	This topic area is comprised of three subtopics, described below.
**Subtopic 1: Understanding violence**	Includes activities, information sharing or group discussion to build understanding of violence (against women) and the different types of violence (e.g., physical, sexual, psychological/emotional, economic); this is often focused on violence against women and girls, but not always, e.g., activities that make connections between the violence men have experienced and the violence they may perpetrate. Also includes activities and group discussion of the risk factors, triggers or root causes of violence and the consequences of violence for individuals, families, and communities; may include discussion of cycles of violence, links between norms of masculinity and violence, or connections between individual and societal or structural violence.
**Subtopic 2: Taking action to prevent VAWG**	Includes activities, information sharing, or group discussion to identify ways to engage individuals, the group, and communities to prevent violence against women and girls, including steps to take (may involve the development of action plans); often includes a broader focus on promoting gender equality and may include discussion of allyship or being an active bystander.
**Subtopic 3: Supporting survivors**	Includes activities, information sharing, and group discussion that focus on ways group members can support women experiencing violence, including unpacking stigma and shame, providing stigma-free support, and reaching out to a person experiencing violence; may include developing personal safety plans.
**Questioning gender norms and roles**	Includes activities, information sharing, and group discussion that define the difference between sex and gender; explore the different roles and behaviors individuals are expected to perform based on their sex or gender identity; and discuss culture and gender socialization, masculinities (more often with men), and gender discrimination and inequality [sometimes, but not often, includes explicit discussion of "norms"].
**Fostering healthy relationships**	Includes activities, information sharing, and group discussion that discuss the characteristics of healthy vs. unhealthy relationships; explore care, honesty, equality, trust, and respect as foundations of healthy relationships; break down what love is and how it manifests; and identify ways to foster love, intimacy, and couple connectedness [often references the importance of communication and may be linked to specific activities on defining good communication and building communication skills]
**Recognizing and balancing power**	Includes activities, information sharing, and group discussion that build understanding of power and different types of power (e.g., over, to, with, within), how power manifests in families and societies, and how to balance power; and discuss status and privilege (who has it and who doesn't).
**Promoting effective communication**	Includes activities, information sharing, and group discussion that discuss the importance of communication, most often with partners (or children), for healthy relationships; explore nonverbal communication, active listening, and empathy; examine different communication styles (i.e., passive, aggressive, and assertive; 'I' statements); and build skills for communicating better.
**Resolving conflict**	Includes activities, information sharing, and group discussion that build understanding of what creates conflict, discuss non-violent ways to de-escalate or resolve conflict, and build conflict negotiation skills [often linked with activities on communication skills and/or anger management activities (for men)]
**Social and emotional skills**	Includes activities, information sharing, and group discussion that focus on identifying different thoughts, feelings, and emotions; discuss how to express certain emotions and manage others (e.g., anger) to avoid or resolve conflict, often with an emphasis on building emotional regulation skills; explore methods to calm down; and may include activities discussing self-esteem and self-confidence [some activities are linked to activities designed to build mindfulness, meditation, breath work, etc. to manage stress]

**Gendered division of unpaid care work**	Includes activities, information sharing, and group discussion that focus specifically on who does the care work within the home (childcare and household tasks); explore the impact of gender norms around caregiving on women and girls (and men and boys); examine the links between unpaid care work and paid work opportunities; and promote a more equitable division of household labor and care
**Sexuality and consent**	Includes activities, information sharing, and group discussion that discuss sex, sexuality and sexual intimacy; explore the importance of consent and what it looks like; and address negotiating safe sex and condom use [often embedded within sessions on SRH more broadly, but not always; consent is sometimes addressed within sexual violence sessions]
**Sexual and reproductive health**	Includes activities, information sharing, and group discussion designed to build participants' sexual and reproductive health (SRH) knowledge and support healthy choices and behavior; depending on the program focus, activities may include understanding the human reproductive system, pregnancy, menstruation, HIV, and other sexually transmitted infections; body mapping; and dispelling myths around SRH [these activities are sometimes provided in conjunction with external experts or health providers, and are sometimes linked with specific activities exploring SRH rights]
**Understanding laws, rights, and policies**	Includes activities, information sharing, and group discussion that provide information and clarity about existing laws and policies (e.g., gender equality and GBV laws, inheritance laws) and rights (e.g., understanding human rights more broadly or SRH rights)
**Parenting and child development**	Includes activities, information sharing, and group discussion that focus on understanding children's needs and the stages of child development; explore fatherhood, motherhood, and co-parenting relationships; build parenting and childcare skills, including promoting positive parenting practices [linked to violence against children and understanding the harmful impacts of violent discipline]
**Making (household) decisions**	Includes activities, information sharing, and group discussion that focus on how to make decisions and (where relevant) discuss the benefits of partners making decisions together that affect the family and/or children (e.g., household finances, family planning, preparing for the birth of a child, child discipline); often, but not always, designed to promote more shared decision-making between men and women within the family [often discussed within the context of household financial decisions]
**Violence against children**	Includes activities, information sharing, and group discussion focused on defining specific types of violence children experience (e.g., violent discipline by caregivers, child sexual abuse); the consequences for children's health and development; how to support children experiencing violence; and/or the intergenerational transmission of violence [sometimes linked to content on parenting and child development]
**Managing Alcohol Consumption**	Includes activities, information sharing, and group discussion that reflect specifically on one's own (or a partner's) alcohol consumption; discuss why people use alcohol, links between alcohol and masculinity, and alcohol as a trigger for violence; explore the consequences of overconsumption (e.g., violence, poor financial management, strained relationships with partners and children); and help participants identify steps to reduce or manage alcohol consumption
**Managing household finances**	Includes activities, information sharing, and group discussion that focus specifically on how finances are managed within the household; explore the challenges of financial mismanagement; discuss the benefits of savings; and build financial skills (e.g., creating a budget or making a savings plan) [often, but not always, complemented with activities that discuss who makes household financial decisions to promote more equitable or shared decision-making practices]
**Mental health and psychosocial needs**	Includes activities, information sharing, and group discussion that discuss the negative impacts of stress and trauma; may include explicit discussion of mental ill health (e.g., depression, suicide, trauma) and its relationship with violence; identify ways to cope with or reduce stress, which may include skills-building (e.g., breath work, relaxation, and meditation); build understanding of participants' psychosocial needs and existing social supports (e.g., social support mapping) [may be linked to activities on emotional regulation]

**Health other**	Includes activities, information sharing, and group discussion that focus on other aspects of health (i.e., not SRH or mental health) including physical health, fitness, ageing, wellbeing, nutrition or accessing general health services; or maternal health (e.g., information to support a healthy pregnancy, including health-seeking and health promoting behavior, including men's support and involvement); during pregnancy); or men's health including discussing men's risk taking and health-promoting behaviors.
