# Supplementary material for: A Scoping Review of IPV Prevention Curricula and Their Implementation in Low- and Middle-Income Countries: Implications for the Next Generation of Programs
Source: Int J Environ Res Public Health. 2026 Jul 29;23(8):989. doi: 10.3390/ijerph23080989 (PMC13512808; doi:10.3390/ijerph23080989)
Supplement: Supplementary file 1 [file ijerph-23-00989-s001.zip › Table S3 Inclusion of key topic areas in curricula by program type.pdf]

Supplemental Table S3. Inclusion of key topic areas in curricula by program type

Topic Area	VAWG (n=25)		SRH (n=13)		ECONOMIC STRENGTHENING (n=10)		FAMILY STRENGTHENING (n=7)	
	# curricula[a] (%)	Avg. proportion of sessions[b]	# curricula (%)	Avg. proportion of sessions	# curricula (%)	Avg. proportion of sessions	# curricula (%)	Avg. proportion of sessions
Addressing violence	24 (96.0%)	39.5%	13 (100%)	28.2%	10 (100%)	14.1%	7 (100%)	13.2%
Understanding violence	*24 (96.0%)*	*25.2%*	*13 (100%)*	*19.4%*	*10 (100%)*	*10.9%*	*7 (100%)*	*10.9%*
Taking action	*21 (84.0%)*	*12.3%*	*9 (69.2%)*	*10.7%*	*5 (50.0%)*	*4.7%*	*2 (28.6%)*	*2.2%*
Supporting survivors	*18 (72.0%)*	*12.0%*	*9 (69.2%)*	*6.6%*	*6 (60.0%)*	*4.8%*	*2 (28.6%)*	*2.7%*
Gender norms and roles	24 (96.0%)	16.9%	12 (92.3%)	14.9%	9 (90.0%)	15.2%	7 (100%)	10.9%
Healthy relationships	22 (88.0%)	14.1%	12 (92.2%)	15.0%	8 (80.0%)	12.5%	5 (71.4%)	72.%
Recognizing & balancing power	20 (80.0%)	7.3%	9 (69.2%)	5.8%	7 (70.0%)	6.0%	6 (85.7%)	7.9%
Effective communication	17 (68.0%)	13.0%	12 (92.3%)	19.3%	8 (80.0%)	15.2%	5 (71.4%)	7.5%
Resolving conflict	15 (60.0%)	4.9%	10 (76.9%)	6.8%	7 (70.0%)	6.9%	4 (57.1%)	6.3%
Social and emotional skills	17 (68.0%)	6.7%	9 (69.2%)	8.8%	4 (40.0%)	6.9%	6 (85.7%)	9.5%
Division of unpaid care work	13 (52.0%)	4.1%	9 (69.2%)	5.5%	9 (90.0%)	8.9%	4 (57.1%)	8.9%
Sexuality and consent	12 (48.0%)	4.9%	10 (76.9%)	13.5%	7 (70.0%)	7.8%	3 (42.9%)	2.9%
SRH	8 (32.0%)	3.3%	13 (100%)	22.9%	6 (60.0%)	12.4%	3 (42.9%)	2.3%
Laws, rights and policies	12 (48.0%)	5.7%	6 (46.2%)	5.6%	3 (30.0%)	2.3%	1 (14.3%)	0.6%
Parenting/child development	5 (20.0%)	2.2%	4 (30.8%)	3.9%	4 (40.0%)	3.3%	7 (100%)	42.0%
Making (household) decisions	4 (16.0%)	1.4%	5 (38.5%)	3.2%	5 (50.0%)	8.4%	4 (57.1%)	5.6%
Violence against children	6 (24.0%)	2.2%	1 (7.7%)	0.5%	3 (30.0%)	4.9%	7 (100%)	19.2%
Managing alcohol	5 (20.0%)	0.9%	5 (38.5%)	6.2%	1 (10.0%)	0.5%	2 (28.6%)	3.0%
Household finances	3 (12.0%)	1.9%	2 (15.4%)	1.0%	5 (50.0%)	8.0%	2 (28.6%)	1.1%
Mental health	4 (16.0%)	1.7%	1 (7.7%)	0.6%	2 (20.0%)	1.5%	2 (28.6%)	3.8%
Health other	3 (12.0%)	0.3%	2 (15.4%)	1.9%	3 (30.0%)	1.3%	1 (14.3%)	0.9%

a Number of curricula of the program type that included the topic area
b Average proportion of sessions including the topic across all curricula of that program type (# curricula of that program type including the topic / total # curricula of the program type)
