# Supplementary material for: A Scoping Review of IPV Prevention Curricula and Their Implementation in Low- and Middle-Income Countries: Implications for the Next Generation of Programs
Source: Int J Environ Res Public Health. 2026 Jul 29;23(8):989. doi: 10.3390/ijerph23080989 (PMC13512808; doi:10.3390/ijerph23080989)
Supplement: Supplementary file 1 [file ijerph-23-00989-s001.zip › Table S4 Inclusion of key topic areas in curricula by population reached.pdf]

Supplemental Table S4. Inclusion of key topic areas in curricula by population reached

Topic Area	MALE & FEMALE (n=27)		MALES ONLY (n=14)		FEMALES ONLY (n=14)	
	# curricula[a] (%)	Avg. proportion of sessions[b]	# curricula (%)	Avg. proportion of sessions	# curricula (%)	Avg. proportion of sessions
Addressing violence	26 (96.3%)	25.1%	14 (100%)	33.2%	14 (100%)	31.8%
Understanding violence	*24 (96.3%)*	*17.9%*	*14 (100%)*	*21.2%*	*14 (100%)*	*20.5%*
Taking action	*15 (55.6%)*	*6.8%*	*11 (78.6%)*	*13.3%*	*11 (78.6%)*	*9.9%*
Supporting survivors	*18 (66.7%)*	*7.3%*	*7 (50.0%)*	*6.4%*	*10 (71.4%)*	*11.7%*
Gender norms and roles	25 (92.6%)	14.6%	14 (100.0%)	17.2%	13 (92.9%)	14.5%
Healthy relationships	25 (92.6%)	15.9%	12 (85.7%)	7.9%	10 (71.4%)	13.0%
Recognizing & balancing power	21 (77.8%)	6.5%	11 (78.6%)	7.4%	10 (71.4%)	6.8%
Effective communication	23 (85.2%)	14.8%	8 (57.1%)	10.6%	11 (78.6%)	16.6%
Resolving conflict	21 (77.8%)	7.0%	7 (50.0%)	4.6%	8 (57.1%)	4.9%
Social and emotional skills	18 (66.7%)	7.3%	13 (92.9%)	9.1%	5 (35.7%)	3.4%
Division of unpaid care work	14 (51.9%)	4.3%	10 (71.4%)	6.6%	11 (78.6%)	8.4%
Sexuality and consent	14 (51.9%)	7.0%	9 (64.3%)	6.2%	9 (64.3%)	8.7%
SRH	15 (55.6%)	7.8%	7 (50.0%)	11.2%	8 (57.1%)	10.8%
Laws, rights and policies	8 (29.6%)	3.3%	5 (35.7%)	3.4%	9 (64.3%)	7.5%
Parenting/child development	9 (33.3%)	7.9%	5 (35.7%)	8.1%	6 (42.9%)	7.7%
Making (household) decisions	11 (40.7%)	5.2%	3 (21.4%)	1.6%	4 (28.6%)	2.6%
Violence against children	8 (29.6%)	2.8%	4 (28.6%)	4.5%	5 (35.7%)	7.4%
Managing alcohol	6 (22.2%)	1.5%	4 (28.6%)	2.0%	3 (21.4%)	4.4%
Household finances	8 (29.6%)	4.4%	2 (14.3%)	0.8%	2 (14.3%)	1.3%
Mental health	4 (14.8%)	1.5%	1 (7.1%)	1.0%	4 (28.6%)	2.7%
Health other	5 (18.5%)	1.4%	3 (21.4%)	0.8%	1 (7.1%)	0.2%

a Number of curricula designed for the population that included the topic area

b Average proportion of sessions including the topic across all curricula of that population (# curricula of that population including the topic / total # curricula of that population)
