# Supplementary material for: A Scoping Review of IPV Prevention Curricula and Their Implementation in Low- and Middle-Income Countries: Implications for the Next Generation of Programs
Source: Int J Environ Res Public Health. 2026 Jul 29;23(8):989. doi: 10.3390/ijerph23080989 (PMC13512808; doi:10.3390/ijerph23080989)
Supplement: Supplementary file 1 [file ijerph-23-00989-s001.zip › Table S5 Curricula adaptation and common source materials (n=55).pdf]

Supplemental Table S5. Curricula adaptation and common source materials (n=55)

Characteristics	Number	Percentage
**Adaptation of entire curriculum**		
Direct adaption of earlier curriculum/program	20	36.0%
'Inspired' by an earlier curriculum/program	14	25.5%
**Common sources of adaptation or inspiration**[a]		
Stepping Stones or Stepping Stones SAMRC	9	16.4%
Program P	4	7.3%
Sisters for Life	4	7.3%
Program H	3	5.5%
**Adaptation of curricula content or activities**		
Curricula citing content adapted from earlier sources	50	90.9%
Minimum number of source materials cited	1	-
Maximum number of source materials cited	11	-
Average number of source materials cited	4.3	-
**Common sources of adapted content**[b]		
Engaging Men and Boys in Gender Transformation	19	34.5%
SASA!	17	30.9%
Program H	11	20.0%
Stepping Stones or Stepping Stones SAMRC	10	18.2%
Rethinking Domestic Violence	9	16.4%
One Man Can	8	14.5%
Program P	8	14.5%
It's All One	7	12.7%
Sisters for Life	7	12.7%

a In total, 13 curricula/manuals were cited as source materials for curriculum adaptation; 10 curricula were cited as source materials 'inspiring' curricula development.
b In total, 87 sources of adapted content were cited.
